# Adenosine deaminase-1 delineates human follicular helper T cell function and is altered with HIV

**DOI:** 10.1038/s41467-019-08801-1

**Published:** 2019-02-18

**Authors:** Virginie Tardif, Roshell Muir, Rafael Cubas, Marita Chakhtoura, Peter Wilkinson, Talibah Metcalf, Rana Herro, Elias K. Haddad

**Affiliations:** 1 Department of Medicine, Division of Infectious Diseases and HIV Medicine, Drexel University, Philadelphia, 19102 PA USA; 2 Genentech, San Francisco, 94080 CA USA; 3 Department of Pathology, Case Western Reserve University, Cleveland, OH 44106 USA; 4 La Jolla Institute for Allergy and Immunology, San Diego, 92037 CA USA

## Abstract

Follicular helper T cells (Tfh) play critical roles instructing, and initiating T-cell dependent antibody responses. The underlying mechanisms that enhance their function is therefore critical for vaccine development. Here we apply gene array analysis identifying adenosine deaminase (ADA) as a key molecule that delineates a human Tfh helper program in proliferating circulating Tfh (cTfh) cells and Germinal Centers Tfh (GC-Tfh). ADA-1 expression and enzymatic activity are increased in efficient cTfh2-17/GC-Tfh cells. Exogenous ADA-1 enhances less efficient cTfh1 and pro-follicular Tfh PD-1+ CXCR5+ cells to provide B cell help, while pharmacological inhibition of ADA-1 activity impedes cTfh2-17/GC-Tfh function and diminished antibody response. Mechanistically, ADA-1 controls the Tfh program by influencing IL6/IL-2 production, controlling CD26 extracellular expression and could balance signals through adenosine receptors. Interestingly, dysfunctional Tfh from HIV infected-individual fail to regulate the ADA pathway. Thus, ADA-1 regulates human Tfh and represents a potential target for development of vaccine strategy.

## Abstract (web summary)

Follicular helper T cells are critical in the generation of the antibody response. Here the authors show that adenosine deaminase is involved in the Tfh program and ensuring the provision of B cell help.

## Introduction

Adenosine deaminase-1 (ADA1, EC 3.5.4.4) is an intracellular as well as an ecto-enzyme (cell surface-bound) of the purine metabolism pathway. ADA-1 exerts its functions through both enzymatic and non-enzymatic mechanisms. The enzymatic function of ADA-1 is achieved by irreversible catabolism of adenosine or 2′-deoxyadenosine into inosine or 2′-deoxyinosine via deamination^1^. In humans, functional mutations of ADA-1 leads to early-onset severe combined immunodeficiency (SCID), which is characterized by the loss of functional T, B, and NK lymphocytes, impaired both cellular and humoral immunity, and an extreme susceptibility to repeated and persistent infections which are often caused by “opportunistic” organisms^2^. The severe immunodeficiency, exemplified by massive T cell and B cell death, could be primarily due to accumulation of high toxic levels of 2′-deoxyadenosine released by the breakdown of DNA during lymphocytes cell death, when differentiation and selection occur in the bone marrow or thymus^1^. In addition to 2′-deoxyadenosine toxicity, high levels of adenosine accumulation due to insufficient enzymatic activity of ADA-1, has been shown to be strongly immunosuppressive. In fact, extracellular adenosine, by binding to adenosine receptor 2a (A2aR) expressed by effector T cell, interferes with TCR signaling pathway by elevating intracellular cAMP and activating protein kinase A (PKA). This leads to the activation of C-terminal SRC (CSK) that diminished the levels of phosphorylated ZAP-70, dampened Ca^2+^ flux and ERK1/2 signaling downstream of TCR activation. Consequently, transcriptional events associated with NFAT, NF-kappaB and AP-1 activation are attenuated^3–5^. Non-enzymatic function of ADA-1 could also account for immune system modulation^6^. As a cell surface-bound enzyme, ADA-1 requires plasma membrane-anchoring proteins. Three ADA-1-anchoring proteins have been described: adenosine receptor 1 (A1R), adenosine receptor 2b (A2bR) and CD26 (dipeptidyl-peptidase IV, DDP4)^7^. Through a mechanism dependent upon its binding to cell surface CD26, ADA-1 can enhance differentiation of naive T cells to effector, memory and regulatory T cells^8^. Moreover, during the immunological synapse formed by DC and T cells, ADA-1 interactions with A1R and A2bR (DC side) and CD26 (T-cells side) have been shown to mediate effective co-stimulatory signals and promote T-cell proliferation and differentiation^9^.

Germinal center Tfh (GC-Tfh) cells found in secondary lymphoid tissues are essential for the generation and maintenance of antibody response. In the past decade, three human blood circulating-Tfh (cTfh) subsets, that share functional properties with GC-Tfh cells, have been described: efficient helpers CD4^+^CD45RA^−^CXCR5^+^CXCR3^−^CCR6^−^; cTfh_2_, CXCR5^+^CXCR3^−^CCR6^+^; cTfh_17_ and less efficient helper CD4^+^CD45RA^−^CXCR5^+^CXCR3^+^CCR6; cTfh_1_^10, 11^. cTfh_2_ and cTfh_17_ are known as efficient helper memory T cells, due to their abilities to elicit strong antibody response following their interaction with memory B cells, whereas their counterpart cTfh_1_ subset, provide less efficient help to B cells where this response is associated with a Th1 signature^12^. Many studies have identified cTfh cells as biomarkers in vaccines and diseases^13–18^ and understanding the underlying mechanisms responsible for their optimal function will provide important information in the design of novel vaccines.

In this study, we have performed co-culture experiments of cTfh cells and GC-Tfh with their autologous B cells^18^ followed by unique gene array analysis to account for genes important in T/B cell cross-talk and have identified ADA-1 as a critical molecule that could be associated with efficient helper cTfh_2–17_ and less efficient cTfh_1_ functions. ADA-1 is expressed in the GC of human tonsils and its pharmacological inhibition impeded GC-Tfh helper function and blunted antibody response.

Mechanistically, ADA acts as an allosteric effector, which controls Tfh helper program by enhancing adenosine affinity for A1R (mainly expressed by T/B cells) and receptor functionality. This leads to controlled CD26/IL-2/IL-6 signaling, in addition of reducing adenosine concentration and therefore metabolite accessibility for other ARs, which balance adenylyl cyclase activity.

Of note, ADA-1/CD26 pathway is impaired in cTfh_2–17_/GC-Tfh from HIV-infected individual. Hence, interfering with ADA-1 pathway may be therapeutically relevant for improvement of Tfh-targeted vaccine strategy.

## Results

### ADA-1 is an immune regulator of human Tfh helper program

**Fig. 1:**
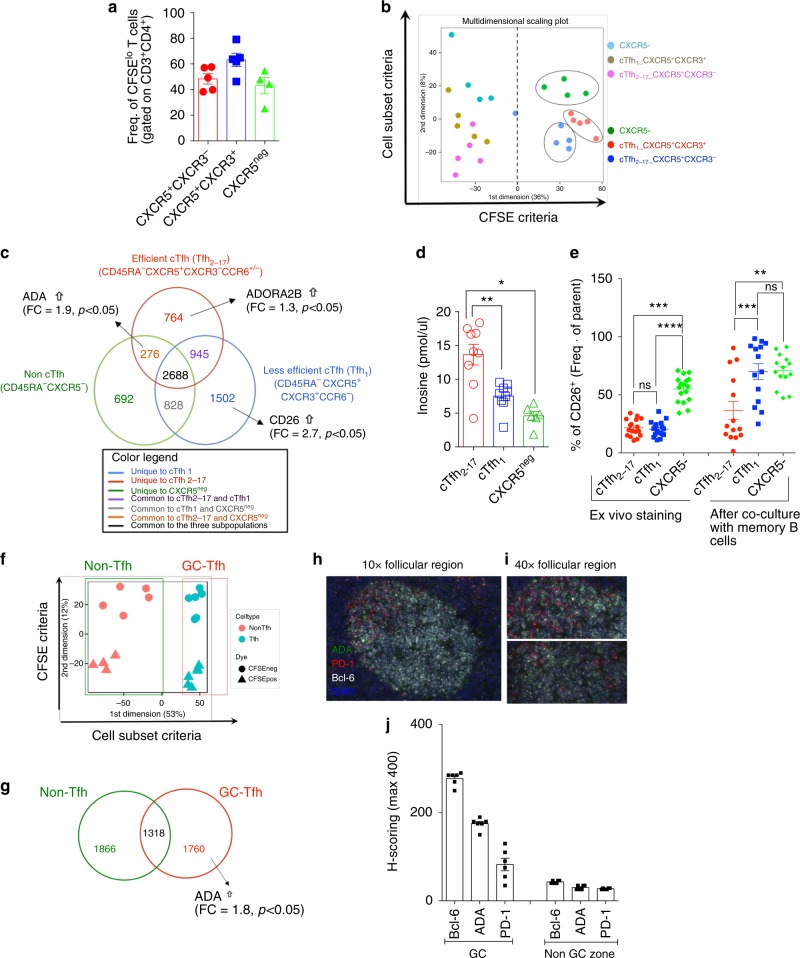
Unique gene array analysis of cTfh and GC-Tfh cells identified B cell helper program. **a** Frequency of CFSE^lo^ T cells in each co-culture set-up. Each T-cell subset is able to proliferate at the same rate (*n* = 5 healthy individuals). **b** Representative multidimensional scaling (MDS) provide a visual representation of the pattern of proximities (i.e., similarities or distances) among a set of objects (memory T-cells subsets). Within the CFSE^lo^ area (right of MDS plot), each subset of T-cells clustered together, suggesting unique gene programs. **c** Venn diagram analysis of non-Tfh CXCR5^−^ population (green) compared to efficient CXCR5^+^CXCR3^+^CCR6^+/−^ cTfh_2-17_ (red) and less efficient CXCR5^+^CXCR3^+^CCR6^+/−^ cTfh1 (light blue). Diagram shows the number of unique and common statistically significant genes based on the above criteria. **d** Inosine level in supernatant of each co-culture set-up (*n* = 9 per group, two-independent experiments, 4 sorts) (Wilcoxon, paired, two-tailed non-parametric *t*-test; *p < 0.05, **p < 0.01, ***p < 0.001, ****p < 0.0001; Mean ± SEM). **e** Ex vivo (*n* = 17 per group, two-independent experiments) and after co-culture (*n* = 14 per group, four-independent experiments) FACS staining for CD26 expression of each T-cells subsets. (ANOVA, paired, two-tailed non-parametric *t*-test; *p < 0.05, **p < 0.01, ***p < 0.001, ****p < 0.0001; Mean ± SEM). **f** Representative MDS is shown as a visual representation of the clustered groups among tonsillar GC-Tfh (red) and non-Tfh (green). Within the CFSE^lo^ group, each T memory group clusters together showing a specific gene profile unique to each subset. **g** Venn Diagram shows the number of unique and common statistically significant genes based on the criteria described in **f**. **h** Representative RNAscope® of a follicular area from tonsil (×10) and **i** (×40). **j** H-scoring for Bcl-6, ADA-1, and PD-1 outside and inside follicular region (*n* = 3 slides per 2 tonsils)

In order to screen for genes that are functionally associated with memory Tfh cell help, we have performed gene expression profiling on efficient helper CXCR5^+^CXCR3^−^ cTfh_2–17_ cells, less-efficient helper CXCR5^+^CXCR3^+^ cTfh_1_ cells and non-Tfh CXCR5^−^ (Supplementary Fig. 1A) following their interactions with autologous memory CD27^+^ B cells (Fig. 1b) in presence of superantigen (SAg) SEB (staphylococcal enterotoxin B), as previously reported^18^. As expected, only efficient helper CXCR5^+^CXCR3^−^ cTfh_2–17_ cells subset provided efficient help to memory CD27^+^ B cells, as shown by the higher IgG/IgA production detected in the supernatant of co-culture (Supplementary Fig. 1C) and the 50/50 ratio of T and B cells rescued after 5 days of co-culture (Supplementary Fig. 1D). Moreover, the Tfh helper program is associated with a unique cytokine profile, i.e. more IL-6 (positive regulator of Tfh function), less IL-2 (negative regulator of Tfh function), less TNF-α and IFN-γ (Th1 cytokines) (Supplementary Fig. 1E–H)^18, 19^. To gain insight into the cTfh_2-17_ cells helper program, we CFSE-labeled sorted cTfh and non-cTfh subsets and re-sorted CFSE^lo^ and CFSE^hi^ T cells from each co-culture for gene array analysis at day 5. CFSE^lo^ cells represent a cell subset that have interacted with B cells and proliferated. Despite the differences amongst the three T-cell subsets in their ability to elicit B cell help (Supplementary Fig. 1C), their capability to proliferate was similar (Fig. 1a), suggesting that B cell help governing mechanisms may be distinct from those that control T-cell proliferation.

Gene expression profiling was performed using Illumina platform, as we previously reported^20–23^. We initially determined multidimensional scaling (MDS) plot (Fig. 1b). MDS analysis provides a visual representation of the pattern of proximities among the memory T-cells subsets. CFSE^lo^ and CFSE^hi^ cells are clustered apart, which is expected as activated to non-activated cells are compared (Fig. 1b). Interestingly, within the CFSE^lo^ cluster, a tight separation of cTfh_2-17_ subset (red) from cTfh_1_ (blue) and CXCR5^−^ (green) subsets (Fig. 1b) is also observed. Differentially expressed genes (DEG) that are common and unique for each subset are identified using Venn diagram analysis (Fig. 1c). The DEG for each subset is obtained by subtracting the CFSE^hi^ (non-dividing cells) expressed genes from the CFSE^lo^ (dividing cells). Accordingly, the resulting DEG list is unique for each T-cell subset and specifically reflects their activated state in the context of B-cell interaction. The Venn diagram shows the number of significant DEGs (fold change (FC) >1.3 or <−1.3; *p* < 0.05) that are unique for each proliferating subset or common between two or three subsets. Our primary interest was to identify DEGs that were unique to responding efficient cTfh_2-17_, less-efficient cTfh_1_ cells and non-cTfh CXCR5^−^. In fact, most of these genes were associated with proliferation, activation markers and other T cell common genes. All genes for all contrasts have been submitted to GEO (GSE99782). We found that adenosine salvage-pathway related genes, i.e., CD26 (DPP4); adenosine deaminase-1 (ADA-1), adenosine receptor 2b (A2bR) were differentially and/or uniquely regulated within each subset (Fig. 1c). Specifically, CD26 was exclusively upregulated within the cTfh_1_ subset (FC~3), while cTfh_2-17_ shows a twofold-increase of ADA-1 (*p* = 9.5 × 10^−5^, *t*-test). ADA-1 was moderately expressed in non-Tfh CXCR5^−^(FC = 1.4; *p* = 0.038, *t*-test) and not significantly altered in cTfh_1_. Selective expression of ADA-1 in cTfh_2-17_ cells was confirmed by measuring ADA-1 enzymatic activity (inosine level) and CD26 expression during co-culture of cTfh/non-cTfh subsets (Fig. 1d, e). Inosine levels were found significantly higher (*p* = 0.0039; Wilcoxon *t*-test) in the supernatant of cTfh_2-17_ co-culture, when compared to those from cTfh_1_ and non-cTfh CXCR5^−^ (Fig. 1e), in line with gene array data and presence of ADA-1 activity in this subset. Moreover, ex vivo CD26 staining showed comparable level between cTfh_2-17_ and cTfh_1_, while higher level in non-cTfh CXCR5^−^. After 7 days of co-culture, CD26 levels were significantly and uniquely increased (*p* = 0.0005; ANOVA *t*-test) on cTfh_1_ subset compared to those on cTfh_2-17_ subset, confirming our gene array data (Fig. 1e).

Similar gene array analysis was also carried out on bona fide Tfh, i.e. GC-Tfh (Supplementary Fig. 2A,B). Briefly, GC-Tfh CD45^neg^CXCR5^hi^PD-1^hi^ and CD45^neg^CXCR5^neg^ non-Tfh were CFSE-labeled and co-cultured 5 days with GC-B cells (CD19^+^ CD38^int^ IgD^neg^ CD319^lo^) (Supplementary Fig. 2C). Only GC-Tfh provided help to GC-B, as shown by IgG production and frequency of rescued GC-B (Supplementary Fig. 3A,B), even though GC-Tfh and non-Tfh cells proliferated at the same rate (Supplementary Fig. 3C). MDS plot shows segregation of proliferating GC-Tfh and non-Tfh cells (Fig. 1f) and Venn diagram analysis revealed that ADA-1 was exclusively expressed within the GC-Tfh subset (FC~2) (Fig. 1g) and not detected in proliferating non-GC-Tfh. Multiplexed in situ hybridization of specific RNA-probes monitoring ADA-1, Bcl-6 and PD-1 expression showed ADA-1 expression at a moderate level (H-scoring 200) inside the GCs of tonsil sections, which were labeled by moderate to high expression of Bcl-6 (H-scoring 250), but at a very low level outside the GC (H-scoring <40) (Fig. 1h–j; Supplementary Figs 4 and 5; Supplementary Tables 1 and 2).

Thus, ADA-1 is expressed and active in GC-Tfh/cTfh_2-17_ cells, respectively, and further represents an indispensable component of human Tfh helper program.

### ADA-1 is required for efficient GC-Tfh response

**Fig. 2:**
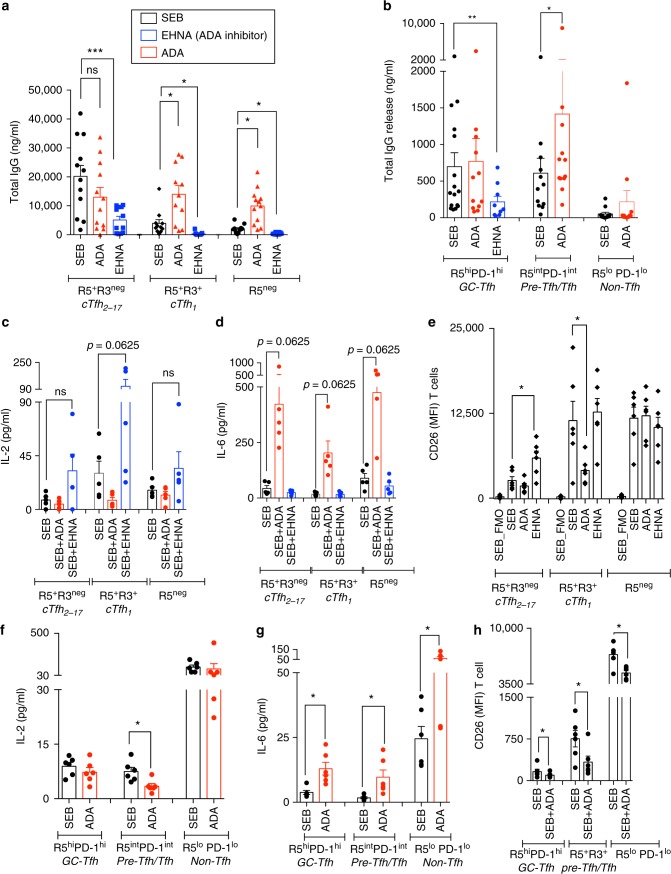
Blocking of ADA impairs cTfh_2-17_ and GC-Tfh helper program. **a** Production of IgG in the supernatants of 7-day co-culture of cTfh and non-cTfh with their autologous memory B cells after inhibition of ADA by EHNA or supplementation with hADA-1 (*n* = 10–11, in four-independent experiments). (ANOVA, paired, two-tailed non-parametric *t*-test; *p < 0.05, **p < 0.01, ***p < 0.001, ****p < 0.0001; Mean ± SEM). **b** Production of IgG in the supernatants of 5-day co-culture of GC-Tfh, pre-Tfh and non-Tfh with their autologous GC B cells after inhibition of ADA by EHNA or supplementation with hADA-1 (*n* = 8–12 per group, in four-independent experiments) (Wilcoxon, paired, two-tailed non-parametric *t*-test; *p < 0.05, **p < 0.01, ***p < 0.001, ****p < 0.0001; Mean ± SEM). **c**, **d** IL-2 and IL-6 production in the supernatants of 7-day co-culture of cTfh and non-cTfh after inhibition of ADA by EHNA or supplementation with hADA-1 (*n* = 5 per group, in two-independent experiments) (Wilcoxon, paired, two-tailed non-parametric *t*-test; *p < 0.05, **p < 0.01, ***p < 0.001, ****p < 0.0001; Mean ± SEM). **e** Extracellular CD26 expression by cTfh and non-cTfh after 7 days of co-culture with or without ADA supplementation or ADA inhibition (*n* = 6 per group, in two-independent experiments). (Wilcoxon, paired, two-tailed non-parametric *t*-test (*p < 0.05, **p < 0.01, ***p < 0.001, ****p < 0.0001). **f**–**g** IL-2 and IL-6 production in the supernatants of 5-day co-culture of GC-Tfh, pre-Tfh and non-Tfh after supplementation with hADA-1 (*n* = 6 per group, in two-independent experiments) (Wilcoxon, paired, two-tailed non-parametric *t*-test; *p < 0.05, **p < 0.01, ***p < 0.001, ****p < 0.0001; Mean ± SEM). **h** Extracellular CD26 expression by GC-Tfh, pre-Tfh and non-Tfh after 5 days of co-culture with or without ADA supplementation (*n* = 6, in two-independent experiments) (Wilcoxon, paired, two-tailed non-parametric *t*-test (*p < 0.05, **p < 0.01, ***p < 0.001, ****p < 0.0001; Mean ± SEM)

**Fig. 3:**
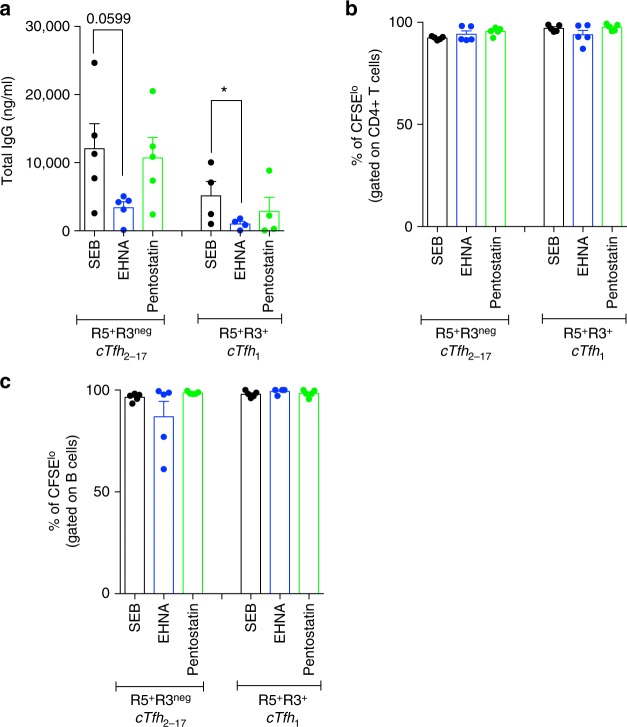
Blocking of ADA with EHNA does not impair T- and B-cell proliferation abilities. **a** Production of IgG in the supernatants of 7-day co-cultures of cTfh and non-cTfh co-in the presence of ADA-inhibitors EHNA or Pentostatin (*n* = 5, in two-independent experiments). (ANOVA, paired, two-tailed non-parametric *t*-test; *p < 0.05; Mean ± SEM). **b** Percentage of CFSE^lo^ T cells after 7-day co-cultures of cTfh subsets with memory B cells in the presence of ADA-inhibitors EHNA or Pentostatin (*n* = 5 per group, in two-independent experiments; Mean ± SEM). **c** Percentage of CFSE^lo^ B cells after 7-day co-cultures with cTfh subsets in the presence of ADA-inhibitors EHNA or Pentostatin (*n* = 5 per group, in two-independent experiments; Mean ± SEM)

We functionally determined, whether ADA-1 was indispensable for cTfh_2-17_ and GC-Tfh efficient helper program using 2 different ADA-inhibitors: EHNA (erythro-9-(2-hydroxy-3-nonyl)adenine); an ADA-1-specific inhibitor^24^, which changes ADA-1 conformation to a closed form^25^ and prevents from binding to its target receptors^26^ in addition to inhibiting its enzymatic activity; and pentostatin (2-deoxycoformycin); a purine transition state analogue of a major intermediate in adenosine catabolic pathway, which causes accumulation of adenosine^27^ and irreversibly inhibit ADA enzymatic activity. Supplementing Tfh/B co-culture with EHNA reduced cTfh_2-17_ (*p* = 0.0002; ANOVA *t*-test) and GC-Tfh (*p* = 0.0039; ANOVA *t*-test) helper functions as shown by significantly lower total IgG production (Fig. 2a, b) and other Ig isotypes (Supplementary Fig. 6A–F) and completely abrogated less efficient cTfh_1_ and non-cTfh CXCR5^−^ helper program (Fig. 2a). This was associated with higher production of IL-2 in all co-cultures (Fig. 2c). However, pentostatin had no effect on IgG production by cTfh_2-17_ (Fig. 3a), suggesting that ADA-1 controls helper program not solely by controlling adenosine concentration, but through yet unidentified mechanisms. Of note, EHNA did not interfere with T/B cells proliferation capacity (Fig. 3b, c), supporting that ADA-1 mediated-Tfh helper program is independent from proliferation program.

In contrast, supplementing T/B co-cultures with exogenous recombinant human ADA-1 significantly (*p < 0.05, ANOVA *t*-test) improves the ability of less efficient cTfh_1_, non-cTfh CXCR5^neg^ and pre-GC Tfh (PD1^int^CXCR5^int^) cells to provide B cell help (Fig. 2a, b). More efficient helper program was associated with higher frequency of rescued CD27^+^ B cells (Supplementary Figs 7A, 8D,E and 9A,B), lower levels of IL-2 release (Fig. 2c–f) and CD26 expression (Fig. 2e–h) (Supplementary Fig. 8A), while inhibition of ADA-1 significantly increased CD26 expression on cTfh_2-17_ (Fig. 2e) (Supplementary Fig. 8A). Of note, CD26 expression levels by memory B and GC-B cells ex vivo (Supplementary Figs 1B and 2C) or after co-culture (Supplementary Fig. 7C,D) were not significant between cTfh and non-cTfh subsets. However, supplementing GC-Tfh or cTfh_2-17_ co-cultures with ADA-1 did not enhance their ability to provide B cell help, suggesting an already optimal function of these cells due to intrinsic ADA-1 presence.

Overall, this falls in line with previous reports, demonstrating a negative effect of IL-2 on Tfh function^18, 28, 29^ and a correlation of CD26 with lack of B-cell functions, probably through the production of IL-2^30, 31^ or ADA-1 sequestration^9, 32^. Interestingly, IL-6, which enhances Tfh function^19, 33, 34^ was significantly elevated with exogenous ADA-1 in peripheral or GC-Tfh co-culture models (Fig. 2d–g), suggesting that ADA-1 could, in part, exerts its positive effect on improving Tfh function through interfering with cytokine network. In this context, ADA-1 also inhibited the production of the Th1 cytokines IFN-γ and TNF-α (Supplementary Fig. 8F–G; Supplementary Fig. 9E,F) but did not significantly alter PD-1 and CD25 expression levels (Supplementary Figs 8A–C and 9C,D). Of note, ADA-induced Tfh helper program was restricted to ADA-1 isoform, as supplementation with ADA-2 isoform (rhCECR1) did not change cTfh_1_ as well as pre-Tfh helper capacities and IL-6 production (Supplementary Fig. 10A–D).

### ADA-1 regulates adenylate cyclase activity

In addition to CD26, ADA-1 can also interact with adenosine receptors 1 (A1R) and 2b (A2bR) and affect its ligand affinity and signaling^9, 35^. Adenosine receptors (AR) belong to a family of G-protein-coupled receptors whose binding of adenosine can either stimulate (A2aR and A2bR) or inhibit (A1R and A3R) adenylyl cyclase activity and cyclic AMP (cAMP) production^36^.

Ex vivo expression of ARs on T/B cells subset (Supplementary Fig. 11A–H) as well as after peripheral T/B interaction (Supplementary Fig. 12A–F) was initially evaluated. A1R, A2aR, A2bR and A3R are expressed at the same levels on cTfh, non-cTfh subsets (Supplementary Fig 11A–D), and on memory B cells (Supplementary Fig. 11E). GC-Tfh and pre-Tfh express nearly identical level of each AR while GC-B cells express higher level of A1R and A3R than A2aR and A2bR (Supplementary Fig. 11H). After 7 days of co-culture (after activation), only A1R level was upregulated on cTfh, non-cTfh (Supplementary Fig. 11A–D) and B-cells subsets (Supplementary Fig. 11E). Interestingly, addition of exogenous ADA-1 or ADA-1 inhibition did not affect ARs expression by either cTfh subsets (Supplementary Fig. 12C,D) or memory B cells (Supplementary Fig. 12E,F).

**Fig. 4:**
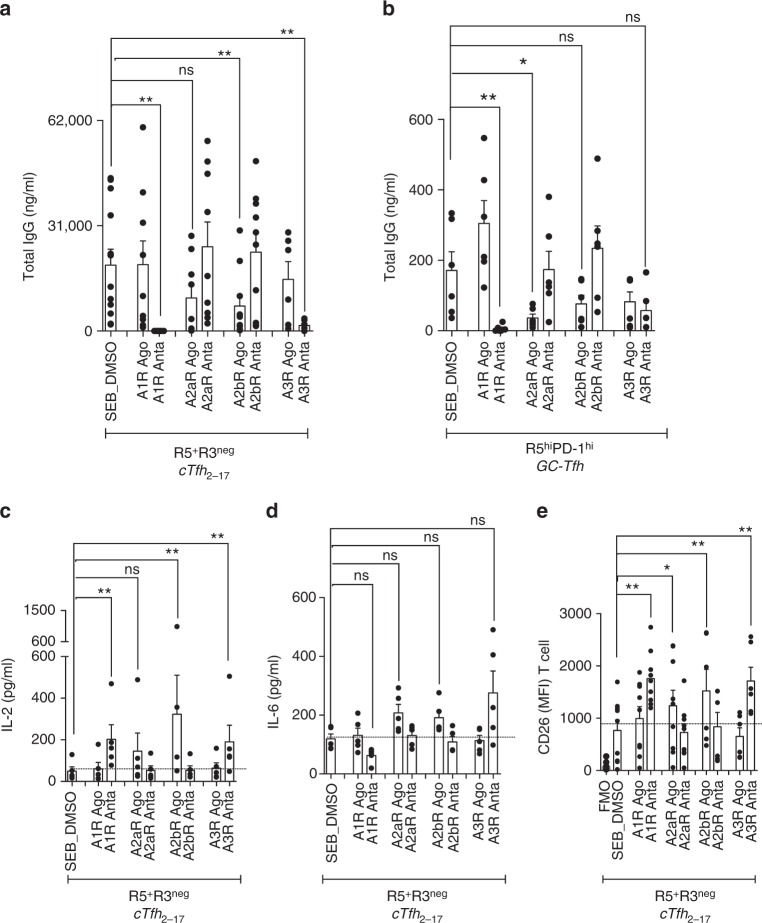
Adenosine receptor signaling fine-tunes Tfh helper program. **a** Production of IgG in the supernatants of 7-day co-cultures of cTfh_2-17_ (CXCR5^+^CXCR3^neg^) in the presence of agonists or antagonists of A1R, A2aR, A2bR and A3R (*n* = 6–10 per group, in four-independent experiments). (ANOVA, paired, two-tailed non-parametric *t*-test; *p < 0.05, **p < 0.01; Mean ± SEM). **b** Production of IgG in the supernatants of 5-day co-culture of GC-Tfh in the presence of agonists or antagonists of A1R, A2aR, A2bR and A3R (*n* = 6 per group, in two-independent experiments). (ANOVA, paired, two-tailed non-parametric *t*-test *p < 0.05, **p < 0.01; Mean ± SEM). **c**, **d** IL-2 and IL-6 production in the supernatants of 7-day co-culture of cTfh_2-17_ (CXCR5^+^CXCR3^neg^) in the presence of agonists or antagonists of A1R, A2aR, A2bR and A3R (*n* = 6 per group, in two-independent experiments) (ANOVA, paired, two-tailed non-parametric *t*-test; *p < 0.05, **p < 0.01; Mean ± SEM). **e** Extracellular CD26 expression by of 7-day co-culture of cTfh_2-17_ (CXCR5^+^CXCR3^neg^) in the presence of agonists or antagonists of A1R, A2aR, A2bR and A3R (*n* = 6–9 per group, in 2–3-independent experiments) (ANOVA, paired, two-tailed non-parametric *t*-test; *p < 0.05, **p < 0.01; Mean ± SEM)

Blocking A1R and A3R signaling with pharmacological antagonists (i.e., blocking the inhibition of adenylyl cyclase activation) completely shutdown helper program of cTfh_2-17_ as demonstrated by significant diminished IgG release (*p* = 0.0030; ANOVA *t*-test) (Fig. 4a), and lower frequencies of rescued memory B cells (Supplementary Fig. 13A), while agonists of these former receptors had no significant effect (Fig. 4a). Only blocking of A1R (not A3R) significantly impaired helper program of GC-Tfh (*p* = 0.0012; ANOVA *t*-test) (Fig. 4b). On the other hand, stimulating A2aR and A2bR signaling with agonists (i.e., induce adenylyl cyclase activation) inhibited GC-Tfh and cTfh_2-17_ helper program, respectively (Fig. 4a, b). This effect was not due to compound-related toxicity, as shown by viability measurement after co-culture assays (Supplementary Fig. 13B), overnight incubation of sorted B cells (Supplementary Fig. 13D,E) or sorted cTfh subsets (Supplementary Fig. 14A) with A1R antagonist or of total PBMCs with either AR agonists or antagonists (Supplementary Fig. 14B,C). Abrogation of cTfh_2-17_ helper program by blocking A1R and A3R signaling was associated with significantly higher production of IL-2 and CD26 T cell expression (Fig. 4c–e) and lower IL-6 production when blocking A1R despite not reaching statistical significance (Fig. 4d).

**Fig. 5:**
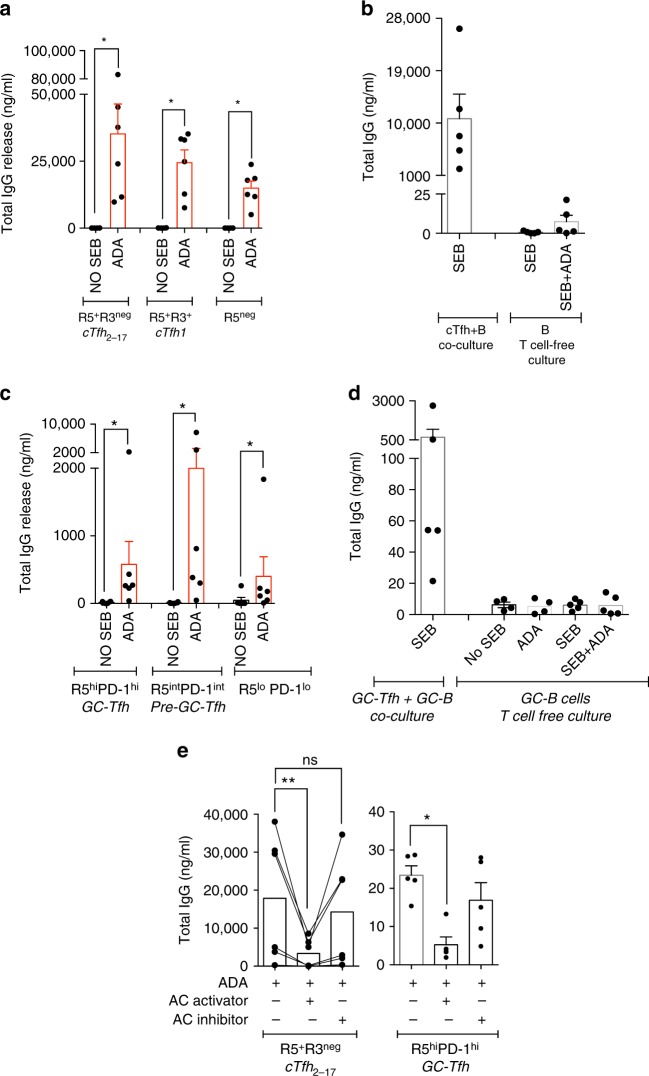
ADA-mediated helper program is blunted by adenylyl cyclase activation. **a** Production of IgG in the supernatants of 7-days co-culture of cTfh and non-cTfh with their autologous memory B cells supplemented with exogenous hADA-1. To note, co-culture assays are performed without any other Sag, i.e. SEB (*n* = 6 per group, in two-independent experiments). (Wilcoxon, paired, two-tailed non-parametric *t*-test; *p < 0.05); Mean ± SEM). **b** Comparison of IgG production in the supernatants of 7-day co-culture of cTfh with their autologous memory B cells and T-cell free culture model (only memory B cells) supplemented with hADA-1 and SEB (*n* = 5 per group, in two-independent experiments; Mean ± SEM). **c** Production of IgG in the supernatants of 5-day co-culture of GC-Tfh, pre-Tfh and non-Tfh with their autologous GC B cells supplemented with exogenous hADA-1. To note, co-culture assays are performed without any other Sag, i.e. SEB (*n* = 6 per group, in two-independent experiments) (Wilcoxon, paired, two-tailed non-parametric *t*-test; *p < 0.05); Mean ± SEM). **d** Comparison of IgG production in the supernatants of 5-day co-culture of GC-Tfh with their autologous GC B cells and T-cell free culture model (only GC B cells) supplemented with hADA-1 and SEB (*n* = 5 per group, in two-independent experiments; Mean ± SEM). **e** Production of IgG in the supernatants of co-culture assay with cTfh_2-17_ (left panel) or GC-Tfh (right panel) performed without adding SEB, but in presence of ADA-1 only, or supplemented with an activator or an inhibitor of adenylyl cyclase (AC) (left panel: *n* = 6 per group, in two-independent experiments; right panel: *n* = 5 per group, in two-independent experiment). (ANOVA, paired, two-tailed non-parametric *t*-test; *p < 0.05, **p < 0.01; Mean ± SEM)

Surprisingly, adding solely ADA-1 into the co-culture assays in the absence of superantigen (SAg) SEB (staphylococcal enterotoxin B) is sufficient for promoting helper program of GC-Tfh, peripheral cTfh and non-cTfh subsets (Fig. 5a–c). However, memory CD27^+^ B cells as well as GC-B cells cultured in presence of ADA-1, but in absence of T cells (T-cell-free culture model), failed to secrete IgG (Fig. 5b–d), suggesting that ADA-1 does not elicit antibody production by B cells independently of T cells and may act as a SAg itself. To further determine whether ADA-1-mediated B cell help could be in part impacted by adenylyl cyclase (AC) activation, we performed co-culture assay in the presence of specific AC activator (Forskolin) or AC inhibitor (NKY80). Co-culture assays were solely performed in the presence of ADA-1, but without SEB, in order to use ADA-1 SAg ability and decipher ADA-1/AC cross-talk. Activation of AC with forskolin significantly abrogated ADA-1-mediated-IgG production by B cells co-cultured with cTfh_2-17_ and GC-Tfh (Fig. 5e), without any forskolin-related-toxicity (Supplementary Figs 13D–G and 14A–C). Inhibition of AC had no effect on cTfh_2-17_/GC-Tfh subsets helper program (Fig. 5e).

All together, these data suggest that ADA-1 fine-tunes AC activity by tightly controlling ARs signaling and is capable of regulating B cell help through interfering with AC/IL-2/CD26 axis.

### CD26 pathway blocks Tfh function by regulating IL-2 release

**Fig. 6:**
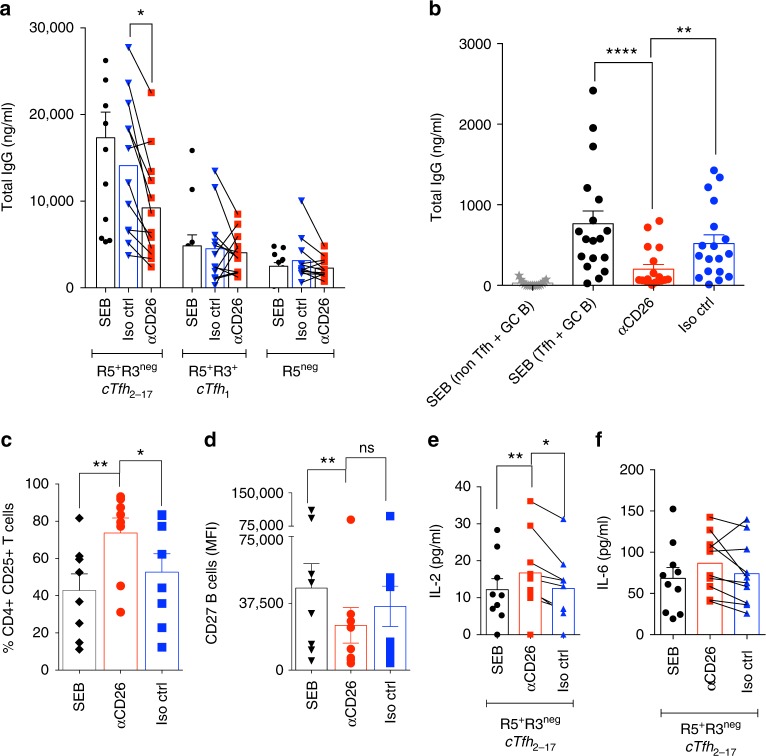
Targeting CD26 pathway blocks Tfh-mediated helper program. **a** Production of IgG in supernatants of the 7-day co-cultures of cTfh and non-cTfh with their autologous memory B cells in the presence of anti-CD26 monoclonal anti-human antibody or its isotype control (*n* = 12 per group, in four-independent experiments). (ANOVA, paired, two-tailed non-parametric *t*-test; *p < 0.05, **p < 0.01; Mean ± SEM). **b** Production of IgG in the supernatants of 5-day co-cultures of GC-Tfh, pre-cTfh and non-Tfh with their autologous GC B in the presence of anti-CD26 monoclonal anti-human antibody or its isotype control (*n* = 17, in four-independent experiments). (ANOVA, paired, two-tailed non-parametric *t*-test; *p < 0.05, **p < 0.01, ***p < 0.001, ****p < 0.0001; Mean ± SEM). **c** Generation of memory B cells (CD27+ B cells). **d** Percentage of CD4^+^CD25^+^ T cells in 7-day co-cultures of cTfh_2-17_ with their autologous memory B cells in the presence of anti-CD26 antibody (*n* = 8 per group, three-independent experiments). (ANOVA, paired, two-tailed non-parametric *t*-test; *p < 0.05, **p < 0.01; Mean ± SEM). **e** Secretion of IL-2 and **f** secretion of IL-6 in the supernatants of 7-day co-culture of cTfh and non-cTfh with their autologous memory B cells in the presence of anti-CD26 antibody (*n* = 9 per group, in two-independent experiments). (ANOVA, paired, two-tailed non-parametric *t*-test; *p < 0.05, **p < 0.01; Mean ± SEM)

It is not entirely clear how ADA-1 interaction with CD26 affects T cell helper program. Therefore, T/B cell co-culture assay were performed in the presence of a monoclonal anti-human CD26 antibody, 134-2C2, known to recognize the ADA-binding site and controls T cells activation through an IL-2/IL-2R-dependent mechanism^37, 38^. To note, CD26 expression by memory B and GC-B cells ex vivo (Supplementary Figs 1B and 2C) or after co-culture (Supplementary Fig. 2B) is not significantly different between cTfh and non-cTfh subsets (Fig. 1e) (Supplementary Figs 1A–7C,D), suggesting that CD26 targeting will preferentially impact T-cells signaling. Targeting CD26 receptor significantly reduced the ability of cTfh_2-17_/GC-Tfh to instruct memory B/GC-B cells to secrete IgG response, respectively (Fig. 6a, b). This coincided with significant reduction in the expression of CD27 on memory B cells, suggesting a decrease in B-cell maturation (Fig. 6c). Investigation of IL-2/IL2R pathway showed that cTfh_2-17_ upregulated CD25/IL-2R expression when targeted for CD26 co-stimulation (Fig. 6d). This was associated with a significant increase in IL-2 secretion (Fig. 6e), while IL-6 release was found unchanged (Fig. 6f). Interestingly, targeting CD26 co-stimulation during co-culture with cTfh_1_ led to increase in IL-2 production (Supplementary Fig. 15A), and significant increase in IFN-γ and TNF-α secretion (Supplementary Fig. 15B,C). CD26 co-stimulation effect on helper program is not due to its dipeptidyl-peptidase activity, as its pharmacological inhibition with aloglipin does not affect helper program of cTfh subsets (Supplementary Fig. 15D).

These results show that CD26 signaling skews co-culture towards IL-2 production, a cytokine that favors a non-Tfh program and further suggests that ADA-1 binding to CD26 might interfere with its IL-2/CD25 cross-talk (Fig. 2c–f) and/or by downregulating CD26 expression (Fig. 2e–h).

### ADA/CD26 axis is impaired during HIV infection

**Fig. 7:**
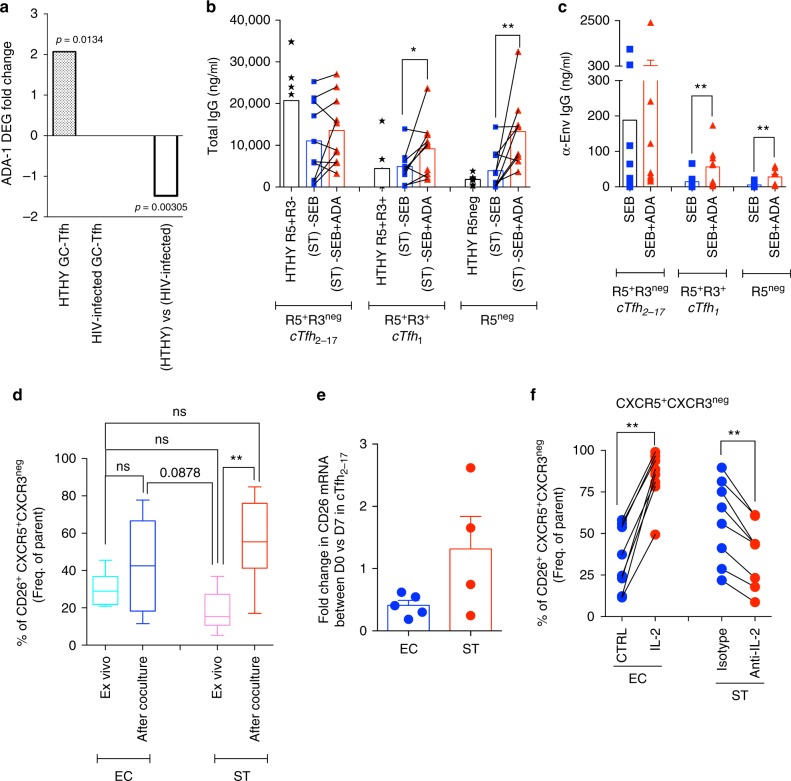
ADA/CD26 axis is impaired during HIV infection. **a** Fold change of ADA-1 gene expression in divided (CFSE^lo^) GC-Tfh after 5 days of co-culture with GC-B cells (*n* = 4 healthy (HTHY) individuals; *n* = 6 HIV-infected individuals; Mean ± SEM). **b** Production of IgG in the supernatants from each ST-cTfh and ST-non-cTfh co-culture after supplementation with ADA-1 (*n* = 6 per group, in two-independent experiments). (Wilcoxon, paired, two-tailed non-parametric *t*-test; *p < 0.05, **p < 0.01; Mean ± SEM). Healthy (HTHY) co-culture have been reported as control. **c** Production of α-Env-IgG in the supernatants from each ST-cTfh and ST-non-cTfh co-culture after supplementation with ADA-1 (*n* = 9 per group, in twoindependent experiments). (Wilcoxon, paired, two-tailed non-parametric *t*-test; *p < 0.05, **p < 0.01; Mean ± SEM). **d** Frequency of ex vivo and 7-day co-culture of CXCR5^+^CXCR3^neg^CD26^+^ T cells subsets in EC and ST individuals (*n* = 8–11, three-independent experiments). (ANOVA, non-paired, two-tailed non-parametric *t*-test; *p < 0.05, **p < 0.01). Whiskers represent Min to Max. **e** Fold change in the relative expression of CD26 mRNA, as assessed by real-time PCR analysis with respect to measurements at day 0. Results from five EC and four ST subjects are shown. Each dot represents the average of two biological replicates with four to six experimental replicates each. Bars represent Mean ± SEM. **f** Frequency of CXCR5^+^CXCR3^neg^CD26^+^ T cells subsets in co-cultures of EC-cTfh in presence of IL-2 (left panel: *n* = 8 per group, two-independent experiments) and from ST-cTfh in presence of anti-IL-2 antibody (right panel: *n* = 7 per group, two-independent experiments). (Wilcoxon, paired, two-tailed non-parametric *t*-test; **p < 0.01; Mean ± SEM)

We have previously shown that GC-Tfh from HIV-infected patient, as well as cTfh_2-17_ from chronic aviremic successfully treated (ST) HIV-infected individuals were dysfunctional^18, 39^. Whether ADA/CD26 pathway was associated with this dysfunction has been evaluated by performing gene expression profiling of sorted proliferating (CFSE^lo^) GC-Tfh cells from HIV-infected and healthy (HTHY) lymph nodes (LN) after their interactions with autologous GC B cells. Proliferating healthy GC-Tfh from LN cells displayed a twofold increase in ADA-1 expression (*p* = 0.0134; *t*-test) (Fig. 7a), which is similar to what we observed with tonsillar GC-Tfh (Fig. 1f–b). However, ADA-1 expression was found unchanged (Fig. 7a) when gene profiling was performed on sorted HIV-infected (CFSE^lo^) GC-Tfh, suggesting that ADA-1 expression is altered in HIV-infected GC-Tfh during GC-B interaction and could negatively impact their ability to provide B cell help. In fact, (ST)-dysfunctional cTfh helper program was improved with exogenous ADA-1, as shown by significant higher production of total IgG and anti-Env IgG in co-cultures started with cTfh_1_ and R5^neg^, respectively (Fig. 7b, c). As we observed before, ADA-1 did not enhance cTfh_2-17_ helper program.

To further delineate the role of ADA-1 during HIV infection, we investigated CD26 pathway in HIV-infected subjects who naturally control HIV infection as in elite controllers (EC) (Supplementary Fig. 16A) and those who control virus replication through anti-retroviral therapy as in ST subjects (Supplementary Fig. 16B). Of note, both groups are virally suppressed (Supplementary Table 3). Dysfunctional (ST)-cTfh_2-17_ showed a significant increase in CD26 expression after co-culture with memory B cells when compared to those from EC (Fig. 7d). In fact, ex vivo expression levels of CD26 on dysfunctional (ST)-cTfh_2-17_ are significantly lower than those on EC or healthy cTfh_2-17_ (Supplementary Fig. 16C,D). The significant increase in CD26 expression levels by (ST)-cTfh_2-17_ suggested dysfunctional ADA-1/CD26 signaling during co-culture. In fact, we found that CD26 mRNA levels at day 7 of co-culture of sorted cTfh_2-17_ subset from ST subjects higher when compared to those sorted from EC co-culture (Fig. 7e). Because CD26 triggered-impairment of helper program is mediated by IL-2/IL-2R signaling axis (Fig. 6d, e) and blocking IL-2 in ST-cTfh_2-17_ restored their ability to provide B cell^18^, we then analyzed whether interfering with IL-2 signaling could down-regulate CD26 expression by ST-cTfh_2-17_. Addition of IL-2 in EC-cTfh_2-17_ co-culture assays significantly increased CD26 expression, whereas blocking IL-2 in ST-ones downregulated CD26 expression (Fig. 7f).

Thus, ADA-1/CD26/IL-2 axis could be partially responsible for polarizing Tfh towards a Th1 program and further for impairing memory B cell help observed during HIV infection.

## Discussion

There has been increasing interest in targeting Tfh cells in the development of new vaccines including HIV vaccines as well as deciphering their role in autoimmune diseases. Evidence on harnessing the function of Tfh in HIV vaccine design was prompted by multiple reports demonstrating that anti-HIV broadly neutralizing antibodies (bnAb), that protect rhesus macaques against SIV challenge^40, 41^, have significant higher rates of mutations than other antibodies, a process mediated by GC-Tfh cells^42^. Moreover, a direct correlation between generation of GC-Tfh cells and the development of tier-2 broad neutralizing antibodies in NHP model has been recently shown^43^. Additionally, the frequency of cTfh_2-17_ PD-1^hi^ cells significantly correlates with the presence of anti-HIV bnAb in humans^44^. Thus, understanding the underlying mechanisms of Tfh biology could have significant impact on pathogenesis of autoimmune diseases^45^ and will provide significant information on how to design better preventive or therapeutic Tfh-targeting vaccine.

**Fig. 8:**
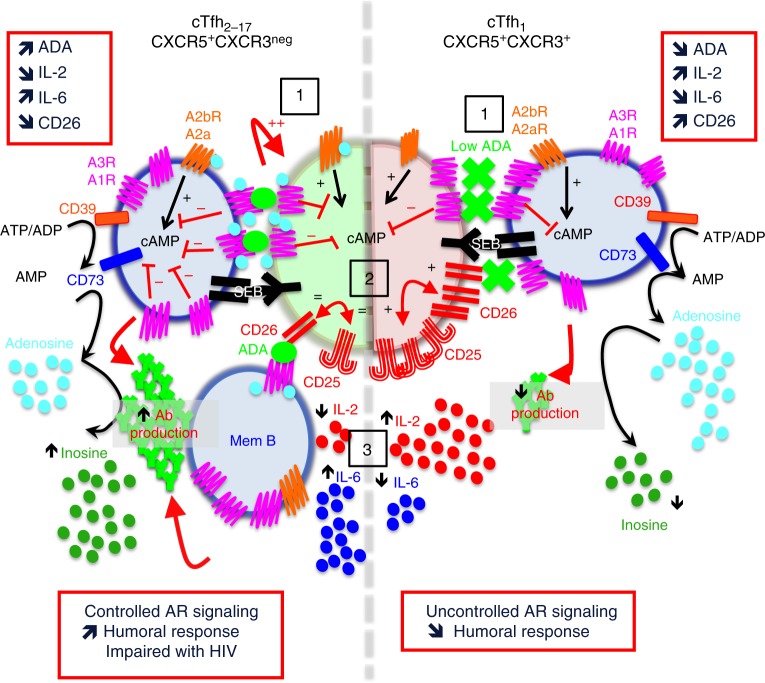
ADA-1 delineates human cTfh subset function – In cTfh_2-17_ subset, ADA-1 by binding to A1R (which is mainly expressed by T/B cells), behaves as an allosteric effector that markedly enhances adenosine affinity for A1R and increases receptor functionality (1) (leading to controlled CD26/IL-2/IL-6 signaling (2–3)), in addition of reducing adenosine concentration and therefore metabolite accessibility for other ARs. Inversely, cTfh_1_ subset does not up-regulate ADA-1 expression, which leads to less efficient memory B cells interaction, more CD26/CD25 cross-talk (2), more IL-2 expression and less IL-6 signaling (3)

In this study, we highlighted ADA-1 as a key molecule that is associated with human Tfh helper function and have identified a molecular mechanism that could explain the differences of B cell help efficiency observed between cTfh_2-17_ and cTfh_1_. Upon interaction with memory B cells, cTfh_2-17_ increases ADA-1 expression, while cTfh_1_ does not (Fig. 8-1), but instead upregulates CD26 expression and enhances production of IL-2 (Fig. 8-2), which in turn skew their helper program towards a Th-1 signature. Our data show indeed that CD26 and IL-2/IL-2R pathways are closely intertwined, which is in line with previous published work^37^ (Fig. 8). On the other hand, ADA-1 expression by cTfh_2-17_ helps to maintain high level of IL-6 which is concomitant with low levels of CD26 expression and IL-2 release, as previously reported^9^ (Fig. 8-3). Thus, ADA-1 functions by providing a favorable cytokine microenvironment promoting Tfh helper program. Of note, reduced expression of CD26 by cTfh_1_, when co-cultured in presence of ADA-1, may partially be due to balanced TNF-α secretion. In fact, neutralization of TNF-α negatively modulates CD26 expression^46^ and our data support this finding. Interestingly, ADA-1 expression was also detected within the GC of healthy individual. Similar to what we observed for cTfh cells, ADA-1 mediated-GC Tfh IgG production is linked to low level of CD26, IL-2 and upregulation of IL-6. Furthermore, we showed that cTfh_2-17_/mem-B and GC-Tfh/GC-B interactions are significantly impaired by ADA-1 inhibition with EHNA. It is worth noting that EHNA is a specific inhibitor of ADA-1, which change ADA-1 conformation to a closed form^25^ preventing ADA-1 to functionally interacts with A1R and A2aR, whose efficient interaction required ADA-1 open form^26^ (Fig. 8-1). In line with these findings, we have demonstrated that A1R, which is highly expressed by both T/B cells after interaction, is critical for cTfh_2-17_/GC-Tfh to elicit its efficient helper program. In fact, blocking A1R signaling, i.e. favoring the AC activity through A2aR/A2bR, induces higher expression of CD26 as well as IL-2 production. Moreover, a role of ADA-1 in controlling AR signaling during GC response cannot be excluded. In fact, recent reports demonstrated significant deleterious effect of A2aR signaling on GC-Tfh differentiation during vaccination^47, 48^. These findings support our data showing that triggering A2aR significantly shutdown GC-Tfh helper program, as does blocking A1R pathway. Overall we proposed a model where ADA-1 by binding to A1R (mainly expressed by T/B cells), may behave as an allosteric effector that could markedly enhance adenosine affinity for A1R (Fig. 8-1) and may increase receptor functionality (i.e., leading to controlled CD26/IL-2/IL-6 signaling), in addition of reducing adenosine concentration (therefore metabolites accessibility for other ARs) and preventing A1R desensitization, as previously^49^. Thus, this may explain why ADA-1-mediated-control of helper program is not abrogated with pentostatin, which only targets enzymatic activity of ADA-1, while EHNA impaired ADA-1 molecular interaction with ARs and CD26. However, further investigations are needed to fully elucidate whether ADA-1 acts independently of controlling adenosine concentration through preferentially ARs molecular interaction or whether both are concomitant. Moreover, an indirect effect of EHNA on phosphodiesterase type 2 (PDE2) could not be excluded, even though our data do not support such a conclusion.

Finally, ADA-1 plays an important role during HIV infection. In fact, ADA-1 expression is missing in GC-Tfh cells from HIV-infected-LN. Interestingly, HIV glycoprotein 120 (gp120) can bind to CD26, and consequently inhibits ADA attachment to it and triggers immunological dysfunction in HIV-infected patients^7, 32, 50^. These findings highlight a pathway, by which HIV infection may interfere with ADA-1 signaling and renders GC-Tfh dysfunctional. More importantly, previous work performed on ADA-1-deficient mice showed that B-cell development was not impaired but LN architectural structure, arguing for a T-cell driven problem for GC formation^51^ and recalling hallmarks of HIV infection. It is also possible that the absence of ADA-1 in HIV-infected-LN could lead to a mis-regulation of adenosine concentration. This condition has been reported in hypoxia where a significant increase in extracellular adenosine levels along with ADA mRNA has been observed in endothelial cells^52^. Moreover, a critical role of hypoxia has been proposed in GC-mediated regulation of antibodies production and affinity maturation^53, 54^. Mis-regulation of hypoxia, as it may happen in the GC of HIV-infected-LN, could lead to the disruption of T/B cells functions causing impairment in the magnitude and quality of antibody, while ADA-1 expression in healthy LN preserves lower levels of adenosine concentrations, regional pattern of hypoxia and functional GCs. Thus, our data demonstrate a beneficial effect ADA-1 on GC function and suggest a role of ADA-1 in instructing antibody affinity maturation within the germinal center, notably by controlling hypoxia. Further work should be performed in order to address this hypothesis.

As mentioned above and as we have demonstrated in this paper, ADA-1 is associated and may have the ability to reprogram the Tfh_1_ signature of cTfh_1_ towards a cTfh_2-17_-like efficient helper program. This skewing of Tfh_1_ response to Tfh_2-17_ has recently been proposed as a strategy to enhance vaccination response, by targeting Tfh functions^55^. On the other hand, in autoimmune infection, such as, adult systemic lupus erythematosus, and rheumatoid arthritis, ADA is found at high level in the serum^56^ and imbalance in the ratio of cTfh_2-17_/cTfh_1_ (cTfh_2-17_ found at a higher frequency) has been reported^45^. Thus, we strongly believe that ADA-1 might represent the two sides of a same coin for immunodeficiency/autoimmunity Tfh-targeted-immunotherapy. Thus ADA-1 function should be fully examined and further investigated as access levels of this molecule may be required for enhancing Tfh function in vaccine development, while absence of this molecule may be required for controlling autoimmune diseases.

## Methods

### Human samples

Blood samples and tonsils from healthy donors, HIV-infected elite controllers (ECs) and HIV-infected chronically treated aviremic subjects (CA) were obtained from the Martin Memorial Health Systems (Florida), the Clinical Research Center, National Institutes of Health (NIH) and the Chronic Viral Illness Service at McGill University Health Centre. The Institutional Review Boards at the relevant institutions approved all procedures, and all participants provided signed informed consent.

### Co-culture assay

PBMCs and/or tonsils from healthy and HIV-infected patients were incubated with fluorochrome-conjugated antibodies for at least 15–20 min at 4 °C or on ice, protected from light. Samples were sorted on a BD™ FACSFusion and/or BD™ FACSAria III for: PBMCs; cTfh_2-17_ cells defined as CD3^+^CD4^+^CD45RA^−^CXCR5^+^CXCR3^−^, less-efficient cTfh_1_ cells defined as CD3^+^CD4^+^CD45RA^−^CXCR5^+^CXCR3^+^; non-cTfh defined as CD3^+^CD4^+^CD45RA^−^CXCR5^−^ and memory B cells defined as CD3^−^CD19^+^CD10^−^CD27^+^ and tonsils; GC-Tfh defined as CD3^+^CD4^+^CD45RA^−^CD25^−^CXCR5^hi^PD-1^hi^, pre-Tfh defined as CD3^+^CD4^+^CD45RA^−^CD25^−^CXCR5^int^PD-1^int^, non-Tfh defined as CD3^+^CD4^+^CD45RA^−^CD25^−^CXCR5^lo^PD^−^1^lo^ and GC-B cells defined as CD19^+^IgD^−^CD38^−+^CD319^lo^. Sorted cTfh or CXCR5^lo^ non cTfh and/or GC-Tfh/pre-Tfh were placed in co-culture with sorted memory B cells and/or GC-B cells respectively at an equal ratio with 100 ng/mL of staphylococcal enterotoxin B (SEB) (Toxin Technology) in RPMI 1640 (Corning) with 10% fetal bovine serum (Access Biologicals) and 1% penicillin/streptomycin (Gibco). To sort CFSE^lo/hi^ cells from the co-culture, total PBMCs and/or tonsils cells were first stained with 1.25 μM CFSE, followed by repeated washing. Cells were then sorted and placed in co-culture, as above, and re-sorted at day 5 for gene array analysis. Alternatively, some co-culture assays were supplemented with either 2.4 μM of human Adenosine Deaminase-1 (Sigma) or 0.5 μg/mL of recombinant human Adenosine Deaminase 2/CECR1 (rhCECR1) (Catalog # 7518-AD) (R&D systems) or with 10 μM of compounds from Tocris: EHNA, inhibitor of ADA-1, or pentostatin or the potent selective A1R agonist (2′-MECCPA), potent selective A1R antagonist (PSB 36), or the potent selective A2aR agonist (PSB 0777), or the potent highly selective A2aR antagonist (SCH442416), or the potent selective A2bR agonist (BAY60-6583), or the potent selective A2bR antagonist (PSB 115), or the potent selective A3R agonist (MRS 5698), or the potent selective A3R antagonist (MRE 3008F20), or the potent selective adenylyl cyclase activator (forskolin), or the potent selective adenylyl cyclase inhibitor (NKY80); or with 0.5 mg/mL of mouse anti-human CD26 (134-2C2) or its isotype control mouse IgM from Southern Biotech. Alogliptin (SYR-322) (A4038 from ApexBio), the inhibitor of dipeptidyl-peptidase activity of CD26, has been used at 5 μM. Cells were kept for 7 days (PBMCs) or 5 days (tonsil) in co-culture followed by flow cytometry analysis. Supernatant and cells were collected at day 7 (PBMCs) or day 5 (tonsil) for subsequent analysis.

### Flow cytometry and antibodies

PBMCs and tonsil cells from HIV-infected or HIV-negative patients were incubated with fluorochrome-conjugated antibodies for at least 15–20 min at 4 °C or on ice, protected from light. The following fluorochrome-conjugated anti-human antibodies were used: CD3 (HIT3α) (dilution: 1/100; Cat. Number: 300324), CD4 (RPA-T4) (dilution: 1/200; Cat. Number: 300518), CD25 (BC96) (dilution: 1/100; Cat. Number: 302608), CD27 (O323) (dilution: 1/100; Cat. Number: 302829), CD38 (HIT2) (dilution: 1/50; Cat. Number: 302532), PD-1 (EH12.2H7) (dilution: 1/100; Cat. Number: 329918 or 329930), CXCR3 (G025H7) (dilution: 1/200; Cat. Number: 353716), CD319 (162.1) (dilution: 1/100; Cat. Number: 331806), CD26 (BA5b) (dilution: 1/100; Cat. Number: 302704), IgD (clone IA6-2) (dilution: 1/100; Cat. Number: 348226), IL7-R (A019D5) (dilution: 1/100; Cat. Number: 351309) and CD19 (HIB19) (dilution: 1/100; Cat. Number: 302216 or 302226) were all from BioLegend. CXCR5 (RF8B2) (dilution: 1/20; Cat. Number: 562781) and Bcl-6 (clone K112-91) (dilution: 1/20; Cat. Number: 561522) were from BD Biosciences and CD45RA (2H4LDH11LDB9) (dilution: 1/200; Cat. Number: IM2711U) from Beckman Coulter. Anti-human adenosine receptor antibodies (dilution: 1/50), i.e., ADORA1 antibody (Cat. Number: orb102041), ADORA2A (Cat. Number: orb15053), ADORA2b (Cat. Number: orb102042), ADORA3 (Cat. Number: orb15062) were obtained from Biorbyt, UK. LIVE/DEAD Fixable Dead Cell Stain (Life Technologies) (dilution: 1/100; Cat. Number: L34957) was used to gate on live cells; and in some cases, both LIVE/DEAD stain and Annexin V (dilution: 1/200; Cat. Number: 561431) (BD Biosciences) were used. Samples were acquired on a BD LSR II.

### Microarray analysis using Illumina BeadChips®

Cells after co-culture were directly sorted into cold RLT buffer (QIAGEN, Frederick, MD, USA) supplemented with 1% beta-mercaptoethanol (BM) (Sigma, St. Louis, MO, USA) and quickly stored at −80 °C. RNA was isolated using a Qiagen’s RNeasy Micro Kit followed by DNase I treatment. Quantification was performed using a NanoDrop spectrophotometer (Thermo Scientific, Wilmington, DE, USA), and RNA quality was assessed using Experion automated electrophoresis system (Bio-Rad, Hercules, CA, USA) with a HeLa RNA-positive control and nontemplate-negative control. RNA was converted into biotinylated cRNA using the Illumina Total Prep-96 RNA amplification kit (Life Technologies). Biotinylated cRNA was normalized and hybridized to the Illumina Human HT-12V4 Expression BeadChips according to the manufacturer’s instruction and quantified using an Illumina iScan System (illumina, San Diego, CA, USA). The data were collected using Illumina genomestudio software. Analysis of the microarray output data were conducted using the r statistical language^21^ and the LIMMA and Combat (batch correction) statistical packages from Bioconductor^57^. Microarrays displaying unusually low median intensity, low variability, or low correlation relative to the bulk of the arrays were discarded from the rest of the analysis. Quantile normalization, followed by a log2 transformation using the LIMMA package, was applied to process microarrays. Subsequently, the LIMMA package was used to fit a linear model to each probe and to perform a (moderated) Student’s *t-*test on various differences of interest^21^. In addition, the LIMMA package from Bioconductor was used to identify differentially expressed genes (DEGs) between CFSE^lo^ vs. CFSE^hi^ for each T cells subsets. Genes with fold change (FC) of ≥1.5 or ≤−1.5 (FC) and *p*-value less than 0.05 were considered significant and were pursued further for validation. Probes that did not map to annotated RefSeq genes and control probes were removed.

### RNAscope® of healthy tonsil

Two human frozen tonsil tissues were sent to ACD (Advanced Cell Diagnostics) for RNAscope® analysis. RNAscope® probes used were the following: Homo sapiens adenosine deaminase (ADA) transcript variant 1 (Cat. No. 490148; NM_000022.3), Homo sapiens programmed cell death 1 (PDCD1) mRNA (aka PD1) (Cat. No. 602028-C2; NM_005018.2), Homo sapiens B-cell CLL/lymphoma 6 (BCL6) transcript variant 1, mRNA (Cat. No. 407258-C3; NM_001706.4). RNA in situ hybridization for ADA, PDCD1 (as PD1) and BCL6 was performed on three-independent slides of each tissues using the RNAscope® LS Multiplex Reagent Kit (Advanced Cell Diagnostics, Inc., Newark, CA) according to the manufacturer’s instructions. Briefly, fresh frozen tissue sections were fixed using 10% NBF at 4 °C for 15 min, followed by a series of dehydration with 50%, 70%, 100 and 100% ethanol. Sections were then pretreated with protease prior to hybridization with the target oligo probes. Preamplifier and amplifier were then hybridized sequentially, followed by TSA-fluorophore reaction. Each channel reacts with a unique fluorophore. Each sample was quality controlled for RNA integrity with a RNAscope® probe specific to POLR2A, PPIB and UBC RNA and for background with a probe specific to bacterial dapB RNA. Specific RNA staining signal was identified as fluorescent, punctate dots. Samples were counterstained with DAPI.

RNAscope® assay results are further evaluated by a semi-quantitative approach used to assign an H-score to regions of interest (ROIs) in samples (outside or inside germinal center). Cells are grouped into 5 bins based on their RNAscope score: 0 = no staining of <1 dot/10 cells; 1 = 1–3 dots/cell; 2 = 4–9 dots/cell, no or very few dot clusters; 3 = 10–15 dots/cell and <10%; 4 => 15 dots/cell and >10% dots are in clusters. Each sample is visually evaluated for the percentage of cells in each bin in a fixed region. The H-score is calculated by totaling the percentage of cells in each bin, according to the weighted formula shown. H-score = [1 × (% cells scored 1) + 2 X (% cells scored 2) + 3 X (% cells scored 3) + 4 X (% cells scored 4)]. The final score ranges from 0 to 400. The H-score includes the sum of individual RNAscope score for each intensity level. By this method, the percentage of cells at each staining intensity level is calculated.

### Total, HIV-specific IgG and Inosine ELISA

For human samples, total and HIV-specific IgG were measured by ELISA on culture supernatant as previously described^18^. Total IgG was detected by coating 96-well Immulon 2HB plates (Thermo Fisher Scientific) with anti-human monoclonal IgG (Mabtech, clone MT91/145) at a concentration of 1 μg/mL in phosphate buffered saline (PBS) overnight at 4 °C. The next day plates were washed three times with wash buffer (PBS + 0.05% Tween 20), and subsequently left to block with wash buffer for 1 h at room temperature. Plates were then washed before the addition of sample and IgG standards at different dilutions, for 1 h at room temperature. HIV Env-specific antibody responses were detected by coating 96-well high-binding half-area plates (Greiner Bio-One) with 1 μg/mL recombinant HIV-1 envelope protein (ProSpec) in PBS and incubating overnight at 4 °C. The next day plates were washed three times with wash buffer (PBS + 0.05% Tween 20), and subsequently left to block with wash buffer for 1 h at room temperature. Plates were then washed before the addition of samples at different dilutions for 2 h at room temperature. For the standard curve, human HIV immunoglobulin was used, and was obtained through the NIH AIDS Research and Reference Reagent Program, Division of AIDS, NIAD, NIH (Cat no. 3957) via Dr. Luiz Barbosa, NABI and National Heart, Lung and Blood Institute. For both assays, following sample incubation and washing, the plates were left to incubate with 1 μg/mL of anti-human IgG-biotin (Mabtech, clone MT78/145) for 1 h at room temperature. The wash step was repeated, and the plates incubated with streptavidin-HRP (Mabtech) for 1 h at room temperature. An extra wash was added to the last wash step before adding 100 μL of TMB substrate (Sigma-Aldrich) to each well until a color change was observed. The reaction was stopped by the addition of 50 μL of 1 M H_3_PO_4_. The OD values were read at 450 nm using a spectrophotometer (SpectraMax Plus, Molecular Devices). For inosine detection, fluorescent (Ex/Em = 535/587 nm) ELISA kit from Raybiotech (68-inosine-100) was used according to manufacturer’s instructions.

### Cytokine and chemokine analysis

Supernatants collected from PBMCs and Tonsils co-cultures were analyzed for chemokine/cytokine levels using Bio-Plex Pro magnetic bead assays (Bio-Rad, Hercules, CA USA). The following human chemokine premixed panels was used: I-309 (CCL1), MCP-1 (CCL2), MIP-1α (CCL3), MCP-3 (CCL7), MCP-2 (CCL8), Eotaxin (CCL11), MCP-4 (CCL13), MIP-1δ (CCL15), TARC (CCL17), MIP-3β (CCL19), 6Ckine (CCL21), MIP-3α (CCL20), MDC (CCL22), MPIF-1 (CCL23), Eotaxin-2 (CCL24), TECK (CCL25), Eotaxin-3 (CCL26), CTACK (CCL27), GM-CSF, GRO-α (CXCL1), GRO-β (CXCL2), ENA-78 (CXCL5), GCP-2 (CXCL6), MIG (CXCL9), IP-10 (CXCL10), I-TAC (CXCL11), SDF-1A+β (CXCL12), BCA-1 (CXCL13), SCYB16 (CXCL16), Fractalkine (CX3CL1), MIF, IL-1β, IL-2, IL-4, IL-6, IL-8, IL-10, IL-16, TNF-α, and IFN-γ. The manufacturer’s protocol was followed. Data were acquired on a Bio-Plex 200 System (using bead regions defined in the Bio-Rad protocol) and analyzed with the Bio-Plex Manager 6.1 software from Bio-Rad.

### Ig isotype analysis

Supernatants collected from PBMCs co-cultures were analyzed for Ig Isotype levels using Bio-Plex Pro magnetic bead assays (Bio-Rad, Hercules, CA USA). The following human Ig isotype premixed panels was used: IgG1, IgG2, IgG3, IgG4, IgM, IgA. The manufacturer’s protocol was followed. Data were acquired on a Bio-Plex 200 System (using bead regions defined in the Bio-Rad protocol) and analyzed with the Bio-Plex Manager 6.1 software from Bio-Rad.

### BioMark

Assays (Primers and Probes) were designed using the Roche Universal Probe Library Assay Design Center (www.universalprobelibrary.com) and were designed to detect multiple transcripts, without respect to isoform prevalence. Cells were sorted directly into PCR plates and immediately frozen. RNA extraction and PCR amplifications were done according to manufacturer recommendations and as we have previously published^18^. Here we show result obtained for CD26 (DPP4; NM 001935.3), forward primer: 5′-GCACGGCAACACATTGAA-3′ and reverse primer: 5′-TGAGGTTCTGAAGGCCTAAATC-3′, probe 20.

### Statistics

All data were analyzed using GraphPad Prism v10. Paired Student’s *t*-test (Wilcoxon) was used when comparing two groups. The Paired multiple *t*-test and non-parametric one-way ANOVA (Friedman) test was used when comparing more than two groups to each other. (*p < 0.05, **p < 0.01, ***p < 0.001, ****p < 0.0001).

### Reporting Summary

Further information on experimental design is available in the Nature Research Reporting Summary linked to this Article.

## Supplementary Information

Supplementary Information

Reporting Summary

## Data Availability

The data that support the findings of this study have been deposited in GEO (Gene Expression Omnibus) with the accession code: GSE99782.
