# Supplementary material for: Adenosine deaminase-1 delineates human follicular helper T cell function and is altered with HIV
Source: Nat Commun. 2019 Feb 18;10:823. doi: 10.1038/s41467-019-08801-1 (PMC6379489; doi:10.1038/s41467-019-08801-1)
Supplement: Supplementary file 1 — Supplementary Information [file 41467_2019_8801_MOESM1_ESM.pdf]

Supplementary Figures 1-16 and Tables 1-3 for manuscript:

Adenosine DeAminase-1 delineates human Follicular helper T cell function and is altered with HIV

Virginie Tardif[1], Roshell Muir[1], Rafael Cubas[2], Marita Chakhtoura[1], Peter Wilkinson[3], Talibah Metcalf[1], Rana Herro[4], Elias K. Haddad[1*].

[1]Drexel University, Department of Medicine, Division of Infectious Diseases & HIV Medicine, Philadelphia, Pennsylvania, United States of America

[2]Genentech, San Francisco, California, United States of America

[3] Department of Pathology, Case Western Reserve University, Cleveland, OH 44106.

[4] La Jolla Institute for Allergy and Immunology, San Diego, California, United States of America

Supplementary Figure 1

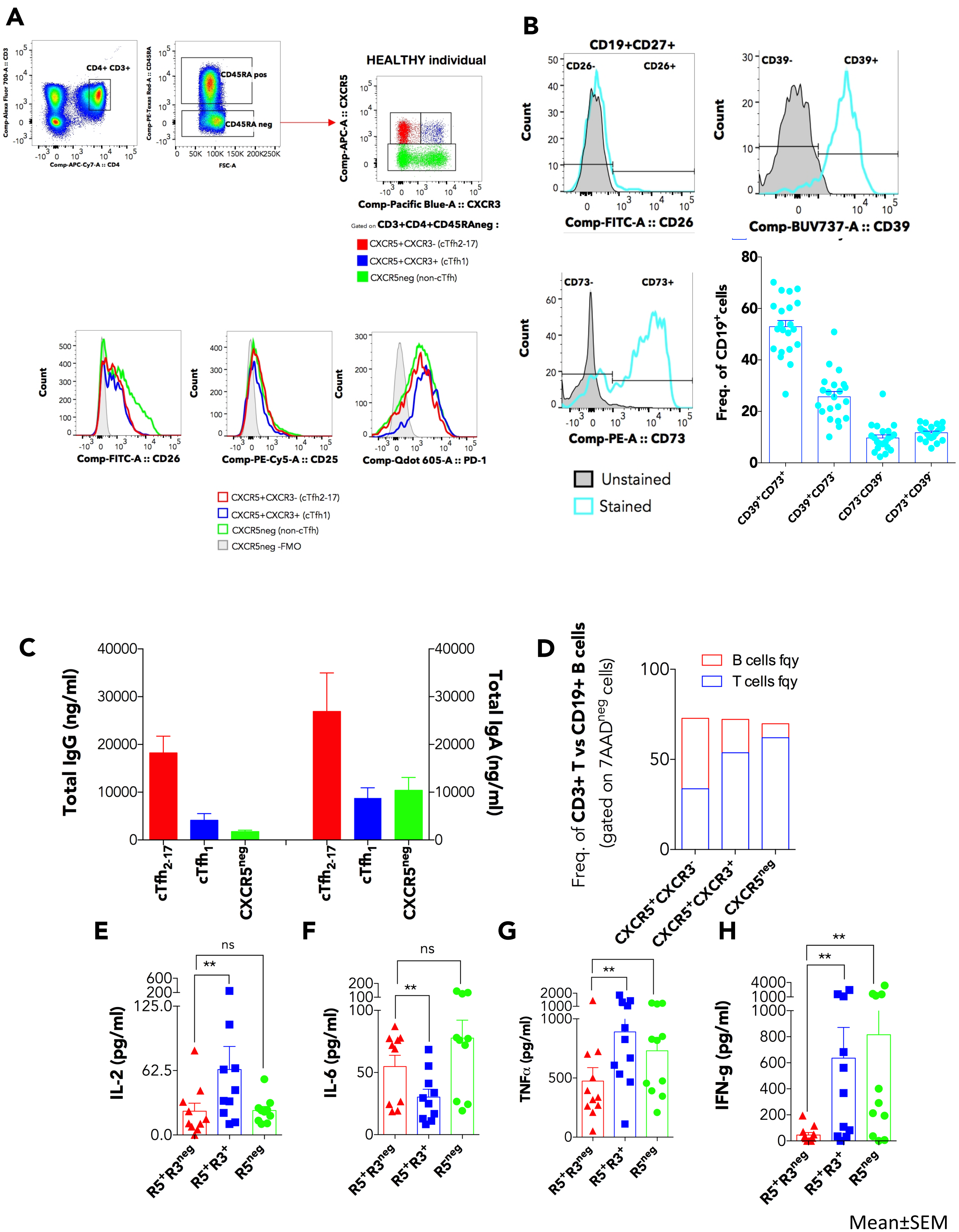

Supplementary Figure 1

(A-B) *Ex vivo FACS representative profile of Tfh cells and memory B cells subsets from PBMCs* **(A)** $CXCR5^{+}CXCR3^{+}CCR6^{+/-}$ $cTfh_{2-17}$ (red); $CXCR5^{+}CXCR3^{+}CCR6^{+/-}$ (blue) and non Tfh $CXCR5^{neg}$ (green) cells subsets, used in the co-culture model, are gated on lived $CD3^{+}CD4^{+}CD45RA^{neg}$ and separated by CXCR5 and CXCR3 staining strategy. Same subset code color is kept in Figure 1 in the main manuscript and Supplementary Figure 16. Bottom histograms show overlaid CD26, CD25 and PD1 expression for each subset, i.e. $cTfh_{2-17}$; $cTfh_{1}$ and $CXCR5^{neg}$ T cells. **(B)** Memory B cells, used in the co-culture assay, are gated out on lived $CD19^{+}CD27^{+}$ cells. Histograms show CD26, CD39 and CD73 expression level by memory B cells $CD19^{+}CD27^{+}$. The majority of the memory B cells expressed both CD73 and CD39.

(C-H) *Molecular baseline of the co-culture model from PBMCs.* **(C)** IgA (right Y-axis) and IgG typical secretion (left Y-axis) in the supernatants of 7-day co-culture of non Tfh $CXCR5^{neg}$ population (green), $cTfh_{2-17}$ (red) and $cTfh_{1}$ (blue) with their autologous memory B cells. Results show significant B cell help by $cTfh_{2-17}$ when compared to the other subsets. **(D)** Ratio of live ($7AAD^{neg}$) T vs B cells in each type of co-culture **(E)** IL-2 (cytokine known to inhibit Tfh function), **(F)** IL-6 (cytokine known to improve Tfh function), **(G)** TNF-α and **(H)** IFN-γ typical secretion in the supernatants of each co-culture at day 7. (ANOVA, paired, nonparametric t-test (*$p < 0.05$), Mean±SEM).

Supplementary Figure 2

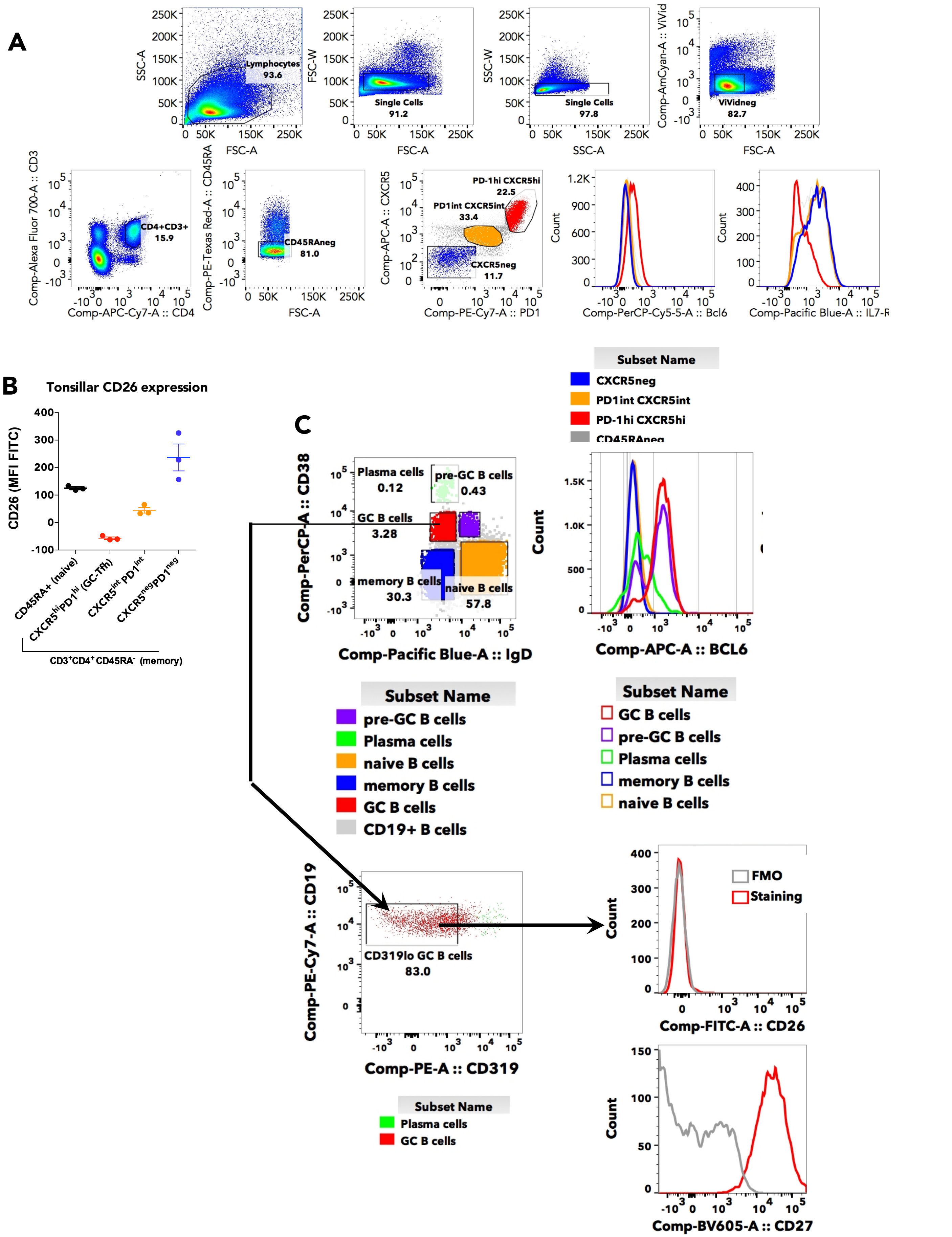

Mean±SEM

Supplementary Figure 2:

Ex vivo FACS representative profile of GC-Tfh, pre-Tfh/Tfh, CXCR5^{neg} and B cell subsets from human tonsil

(A) CXCR5^{hi}PD-1^{hi} (GC-Tfh) (red), CXCR5^{int}PD-1^{int} (pre-Tfh/Tfh) (orange), CXCR5^{neg} (blue) subsets, used in the co-culture model, are gated lived CD3^{+}CD4^{+}CD45RA^{neg} and separated by CXCR5 and PD-1 staining strategy. Of note, each T cell population has been sorted from CD25^{neg} population (not shown). Histograms show overlaid Bcl-6 and IL7-R expression for each subset, i.e. GC-Tfh; pre-Tfh/Tfh and CXCR5^{neg} T cells. **(B)** Graph showing CD26 MFI for each subset, i.e. GC-Tfh; pre-Tfh/Tfh and CXCR5^{neg} T cells. **(C)** GC-B cells, used in the co-culture assay, are gated out on lived CD19^{+} B cells and separated by IgD and CD38 staining strategy. GC-B cells (red) are IgD^{neg}CD38^{int}CD319^{lo}Bcl6^{+} and histogram show *ex vivo* CD26 (top) and CD27 (bottom) expression by GC-B.

Supplementary Figure 3

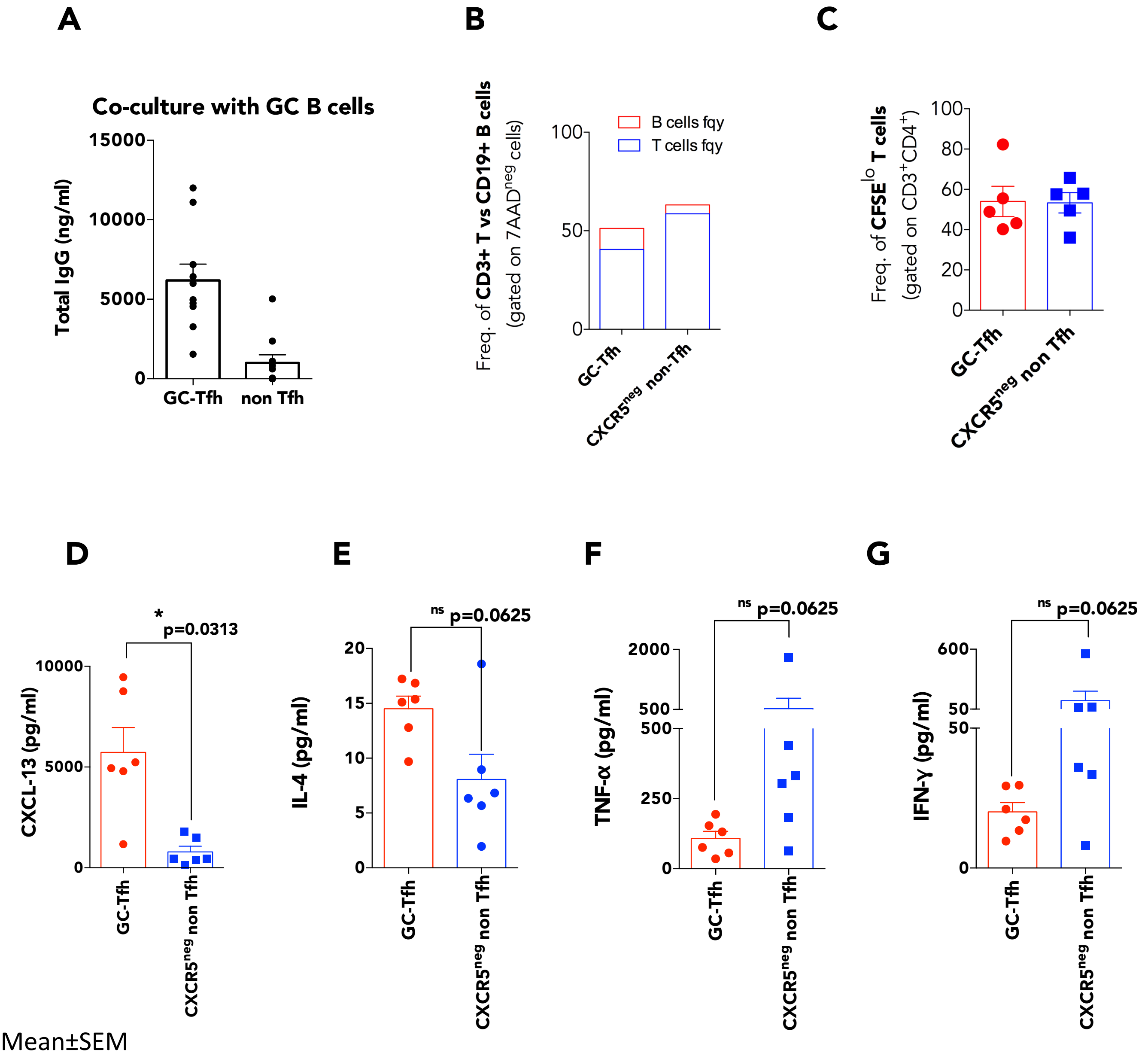

Supplementary Figure 3: *Molecular baseline of the co-culture model from Tonsil cells.* **(A)** Typical IgG production in the supernatants of day 5 co-culture showing the ability of CXCR5^{hi}PD-1^{hi} (GC-Tfh) to elicit antibody production. **(B)** Ratio of live (7AAD^{neg}) T vs B cells in GC-Tfh and non Tfh CXCR5^{neg} co-culture assays. **(C)** Frequency of CFSE^{lo} T cells harvested after 5 days of co-culture showing similar proliferation capacity in both GC-Tfh and non-Tfh cells. **(D)** CXCL-13, **(E)** IL-4**, (F)** TNF-α, and **(G)** IFN-γ secretion in the supernatant of each co-culture at day 7. Of note, each T cell population has been sorted from CD25^{neg} population (not shown). (Wilcoxon, paired, nonparametric t-test (*$p < 0.05$; Mean±SEM).

Supplementary Figure 4

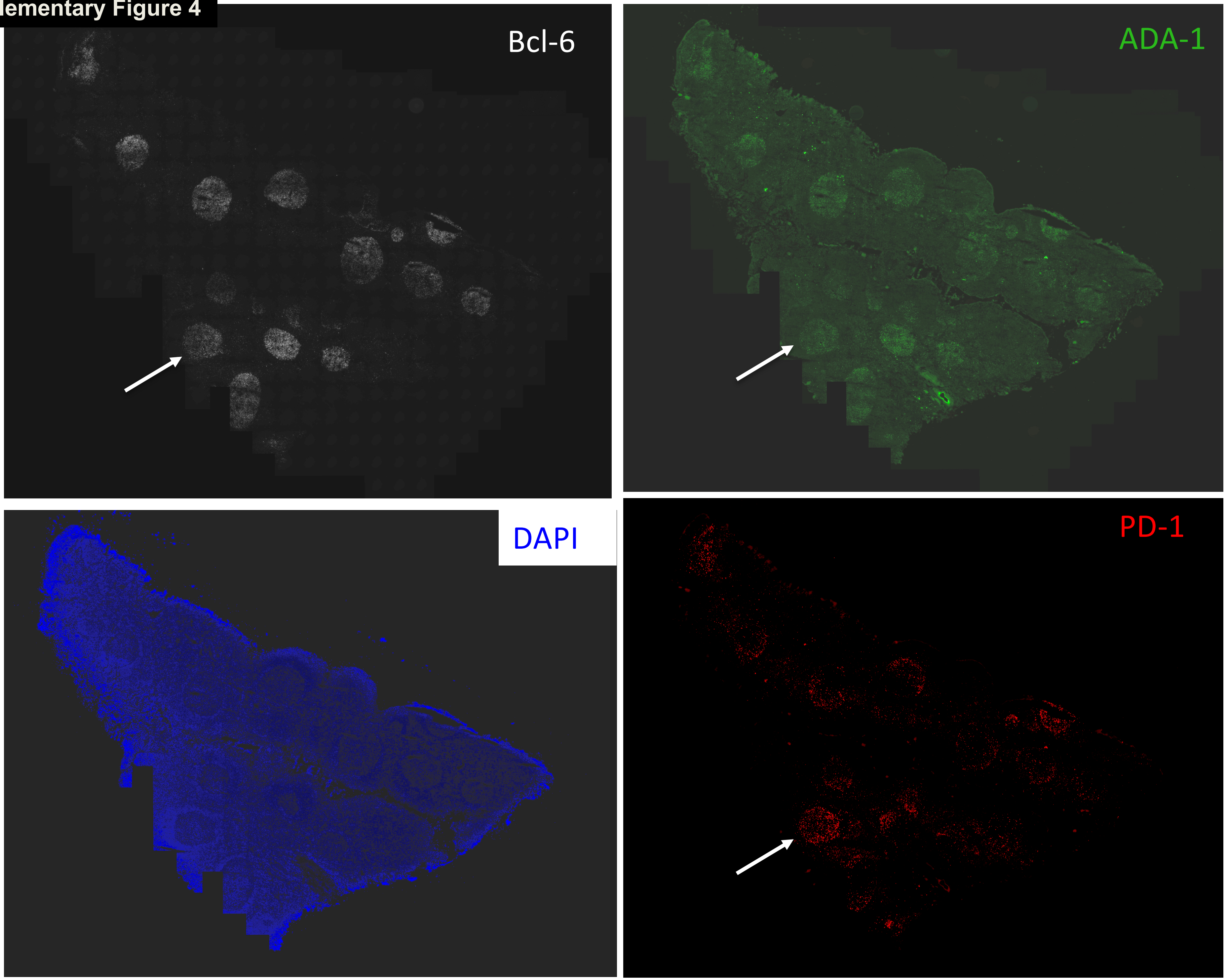

Supplementary Figure 4: *RNAscope® of each channel representing a full piece of tonsil*

Each channel (Bcl-6 -ADA-1-PD-1-DAPI) is shown separately. Bcl-6 is used to delineate GC area. Arrow shows follicular area zoomed out in the following Supplementary Figure 5.

Supplementary Figure 5

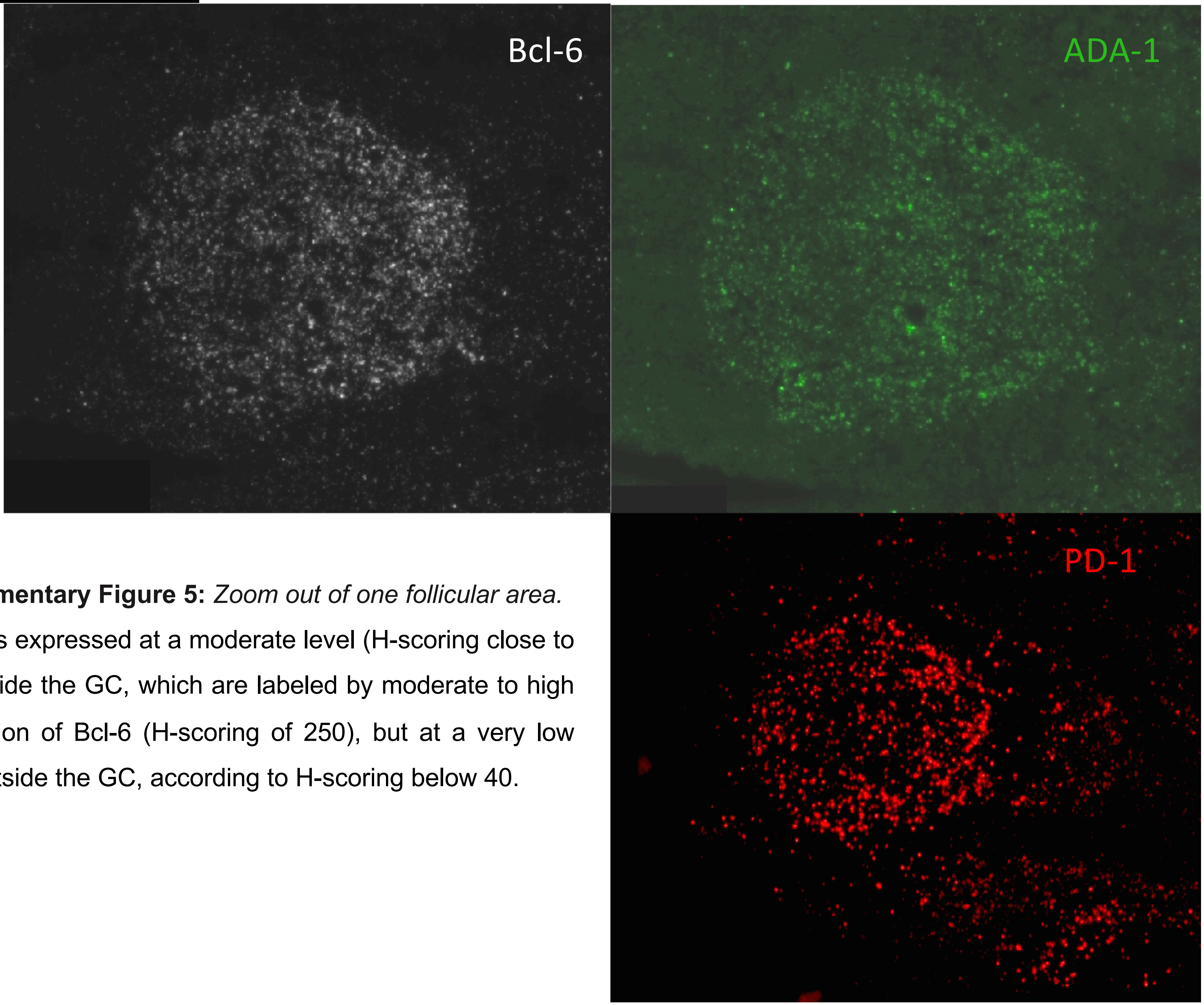

Supplementary Figure 5: *Zoom out of one follicular area.* ADA-1 is expressed at a moderate level (H-scoring close to 200) inside the GC, which are labeled by moderate to high expression of Bcl-6 (H-scoring of 250), but at a very low level outside the GC, according to H-scoring below 40.

Supplementary Figure 6

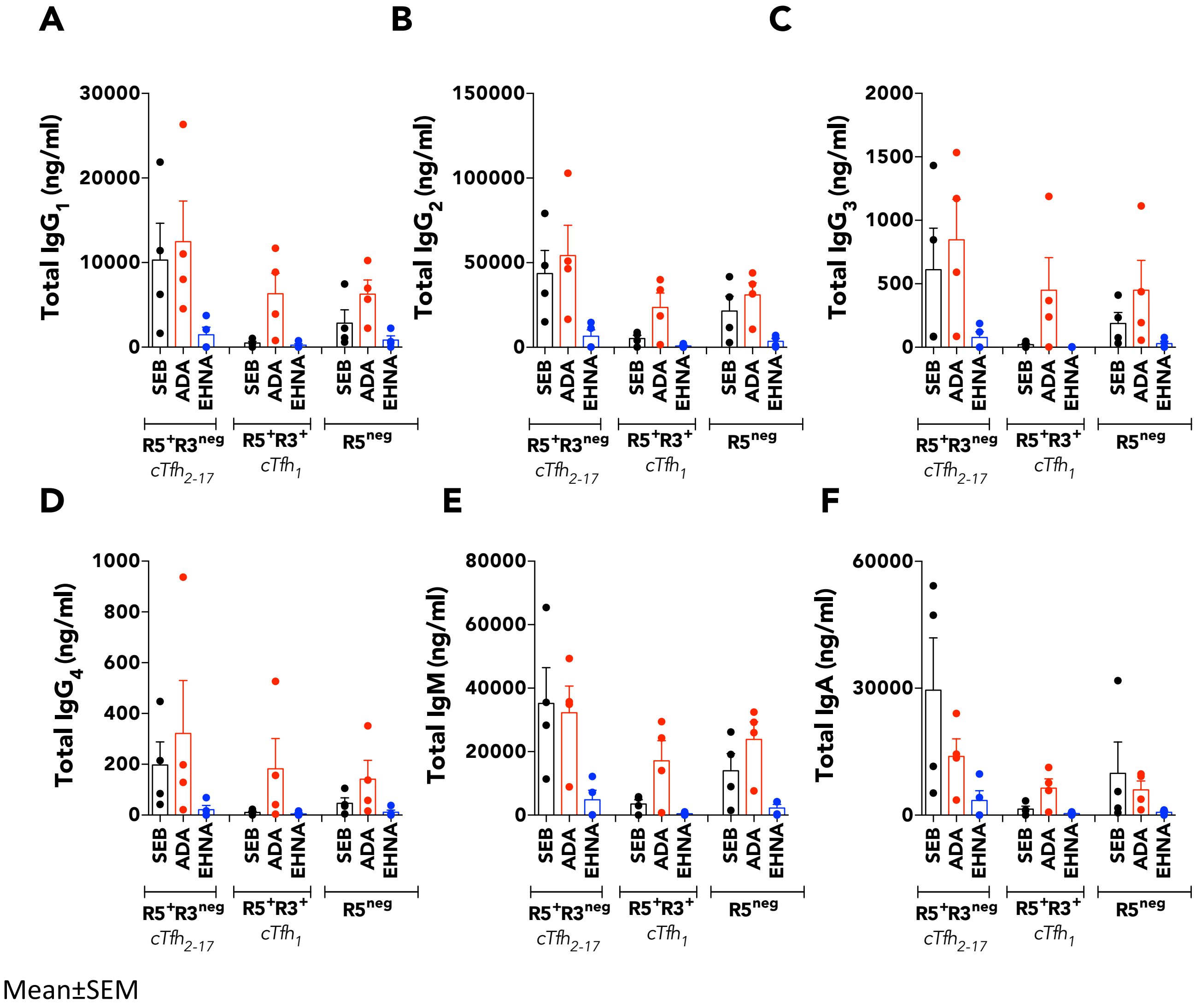

Supplementary Figure 6: *Assessment of Ig Isotype in the supernatant of cTfh co-culture.*

(A) IgG1, **(B)** IgG2**,** **(C)** IgG3, **(D)** IgG4, **(E)** IgM **(F)** IgA secretion in the supernatant of each co-culture at day 7.

Supplementary Figure 7

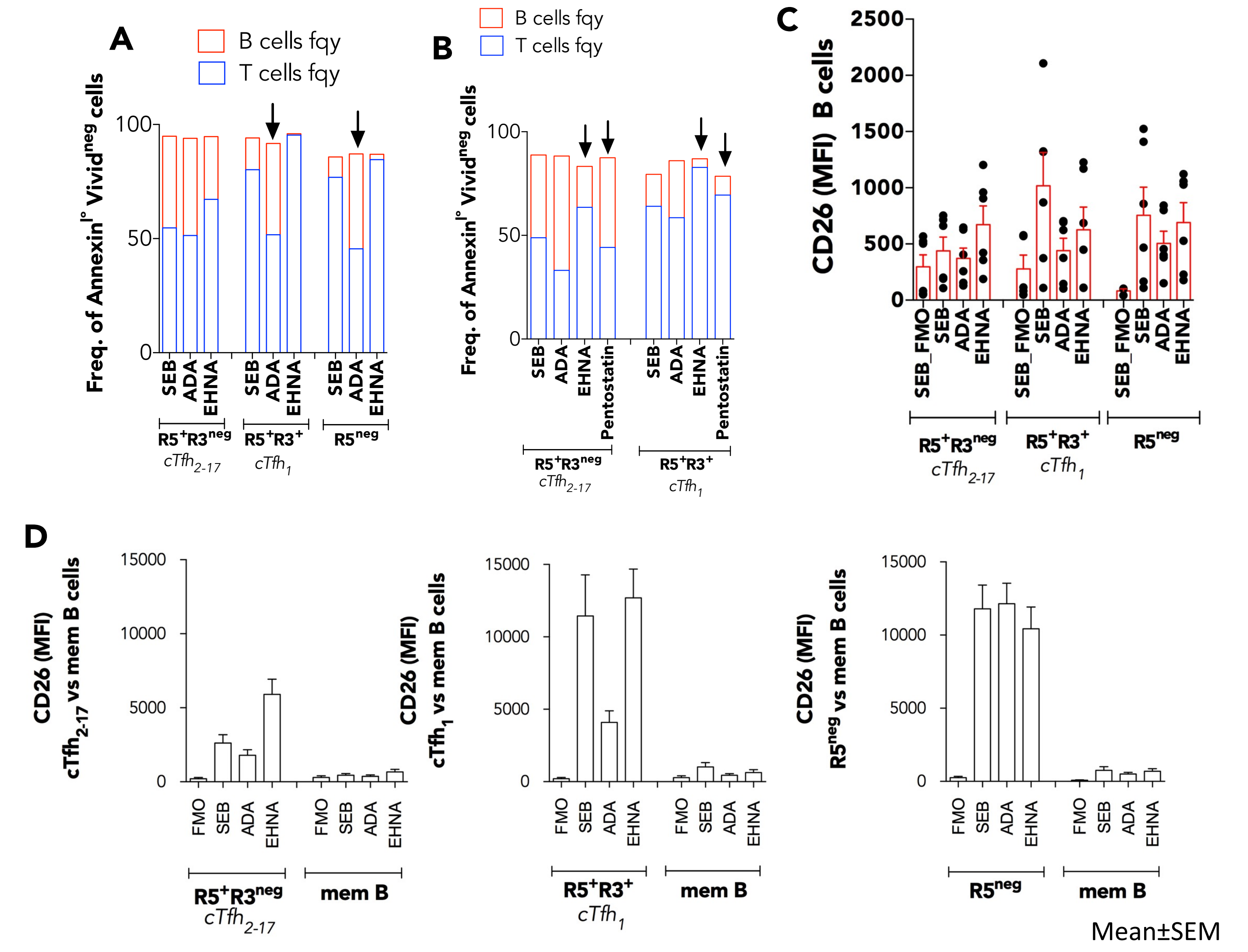

Supplementary Figure 7: *Viability and CD26 expression by T and B cells subsets after co-culture assays from PBMCs*

(A) Ratio of live (Annexin^{neg}) T vs B cells at day 7 of co-culture with ADA or its inhibitors EHNA or **(B)** pentostatin for each cTfh and non cTfh subsets. **(C)** CD26 expression in MFI by memory B cells after co-culture with either cTfh or non-cTfh subsets. **(D)** Graphs show a comparison of CD26 expression by cTfh and non-cTfh subsets versus memory B cells from each respective co-culture model. Memory B cells express after co-culture a very low level of CD26 compared to T cells subsets.

Supplementary Figure 8

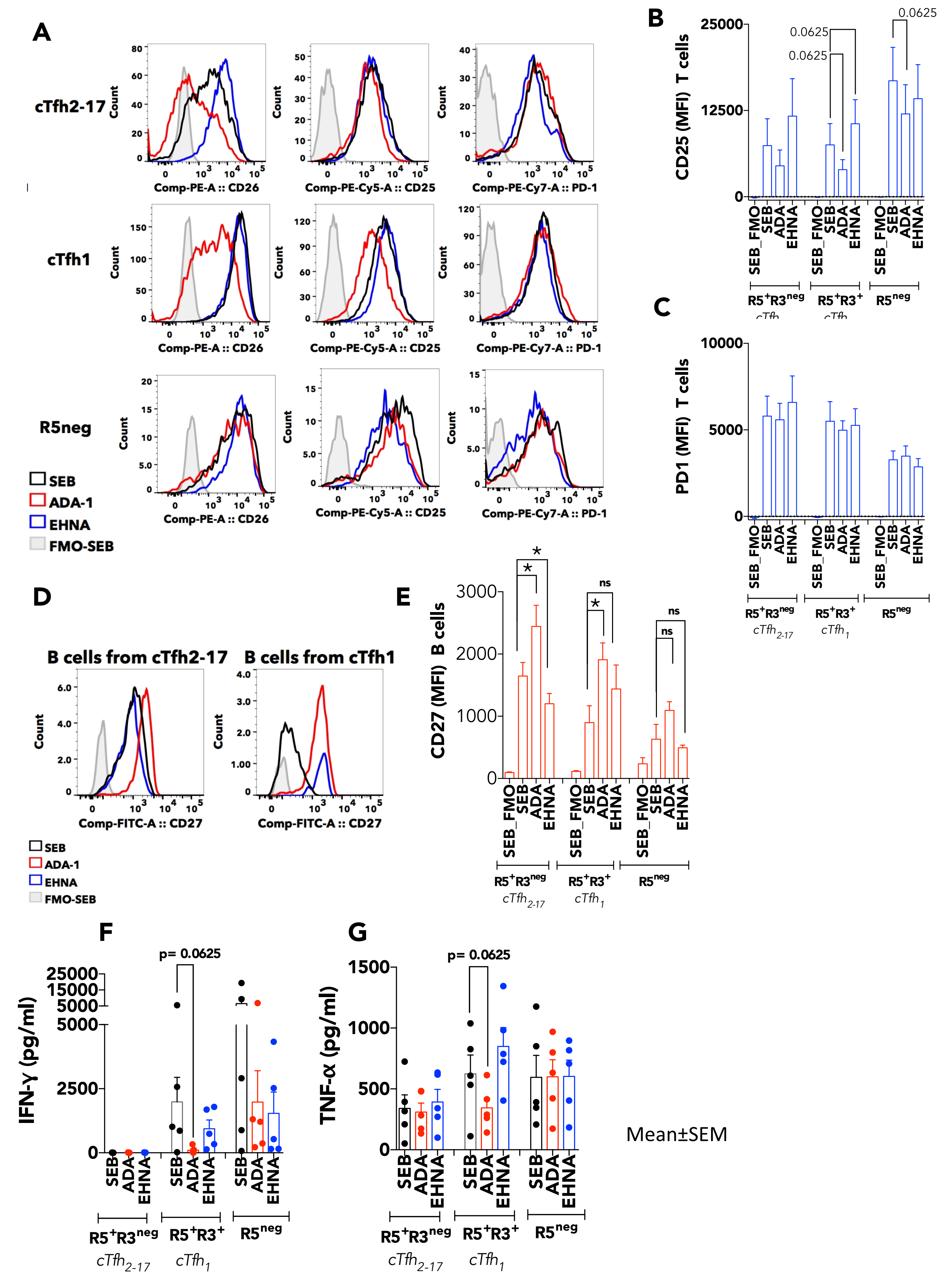

Supplementary Figure 8: *Phenotype and cytokine baseline of the co-culture model from PBMCs supplemented with ADA-1 or its inhibitors.*

(A) FACS representative overlays of CD26, CD25 and PD-1 expression by each cTfh and non-cTfh subsets after co-culture supplemented or not with ADA or its inhibitor. **(B)** CD25 expression (MFI) on T cells in co-culture of cTfh and non cTfh subsets supplemented or not with ADA or its inhibitor. **(C)** PD-1 expression (MFI) on T cells in co-culture of cTfh and non-cTfh subsets supplemented or not with ADA or its inhibitor. **(D)** FACS representative overlays of CD27 expression by memory B cells after $cTfh_{2-17}$ or $cTfh_1$ interaction in co-culture supplemented or not with ADA or its inhibitor. **(E)** CD27 expression (MFI) on B cells in co-culture with cTfh and non-cTfh subsets supplemented or not with ADA or its inhibitor, EHNA**.** **(F)** IFN-γ, **(G)** TNF-α secretion in the co-culture supernatants of cTfh and non cTfh subsets supplemented or not with ADA or its inhibitor, EHNA. (ANOVA, paired, nonparametric t-test (*$p < 0.05$), Mean±SEM).

Supplementary Figure 9

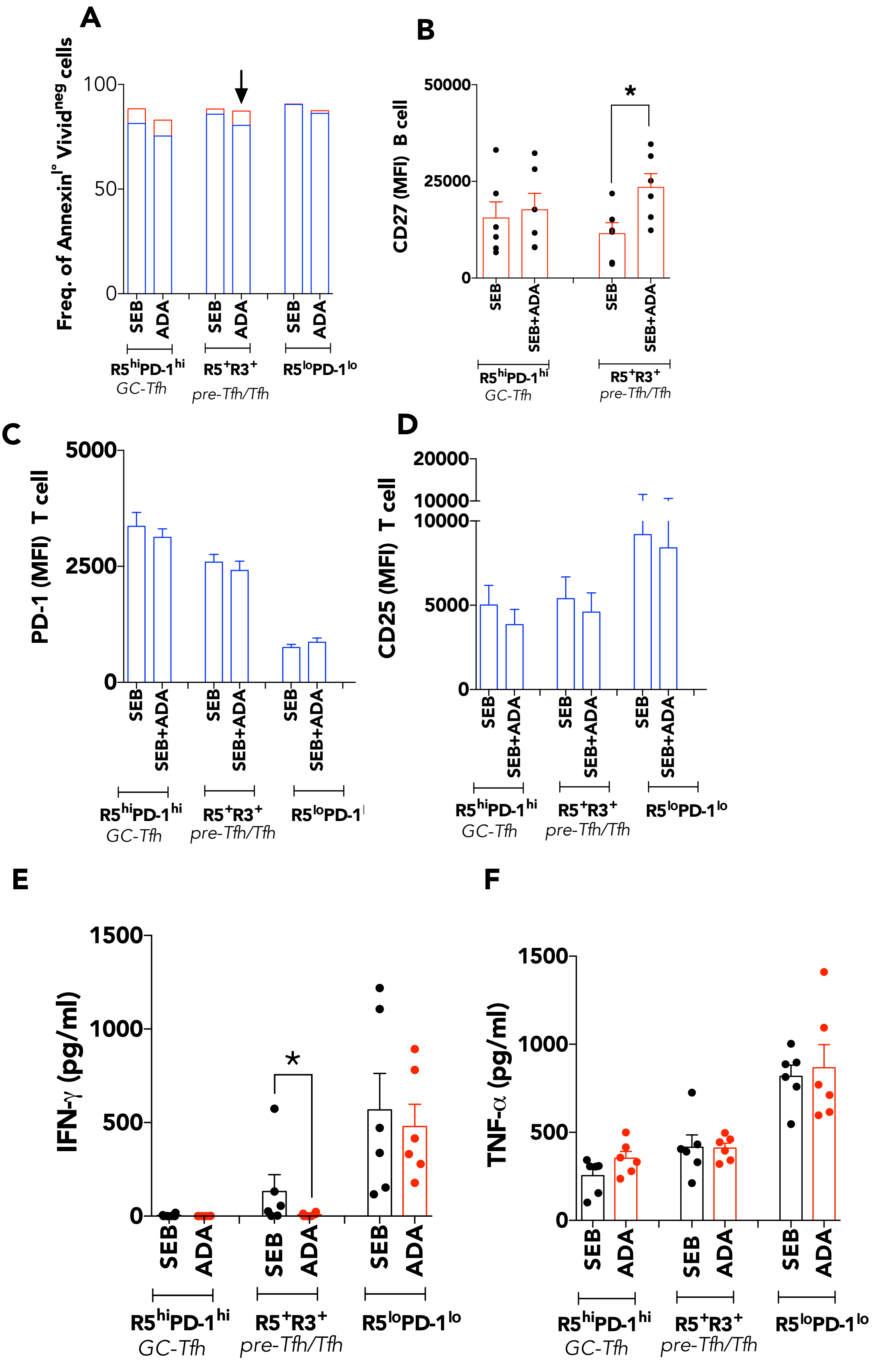

Mean±SEM

Supplementary Figure 9: *Phenotype and cytokine baseline of the co-culture model from tonsil supplemented with ADA-1.*

(A) Ratio of live (Annexin^{neg}) T vs B cells at day 5 of co-culture of GC-Tfh, pre-Tfh or CXCR5^{neg} in the presence or absence of ADA. **(B)** CD27 expression (MFI) on B cells in co-culture with GC-Tfh and pre-Tfh subsets supplemented or not with ADA. **(C)** PD-1 expression (MFI) on T cells in co-culture of GC-Tfh, pre-Tfh or CXCR5^{neg} and supplemented or not with ADA. **(D)** CD25 expression (MFI) on T cells in co-culture of GC-Tfh, pre-Tfh or CXCR5^{neg} supplemented or not with ADA. **(E)** IFN-γ, **(F)** TNF-α secretion in the co-culture supernatants of GC-Tfh, pre-Tfh or CXCR5^{neg} in the presence or absence of ADA. (Wilcoxon, paired, nonparametric t-test (*$p < 0.05$), Mean±SEM).

Supplementary Figure 10

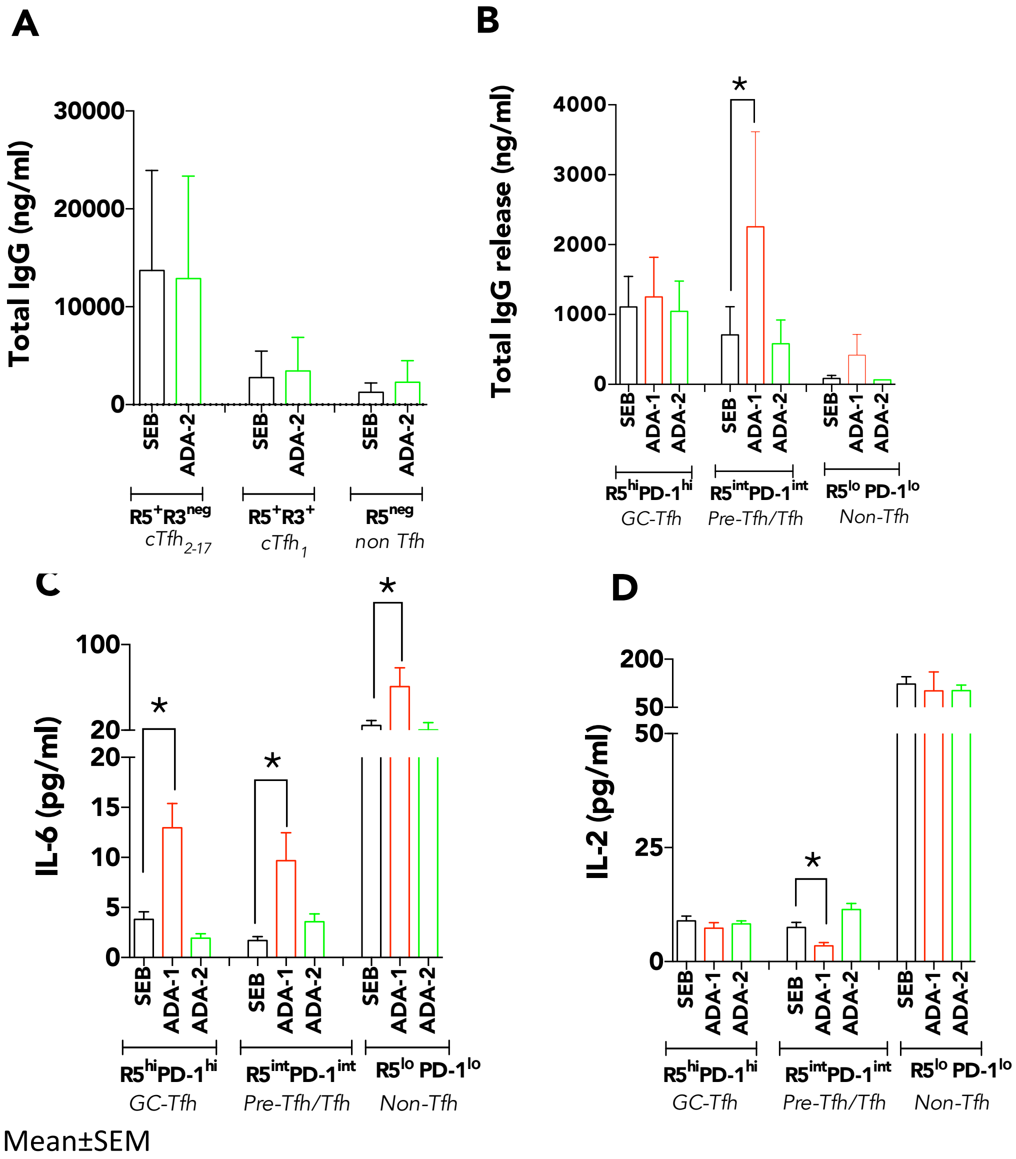

Supplementary Figure 10: *Effect of isoform ADA-2 (CECR-1) on co-culture assay from PBMCs and tonsil.*

(A) Production of IgG in the supernatants of 7-days co-culture of cTfh and non-cTfh subsets with their autologous memory B cells supplemented with exogenous recombinant ADA-2. (n=3 per group). **(B)** Production of IgG in the supernatants of 5-days co-culture of GC-Tfh, pre-Tfh and non-Tfh subsets with their autologous GC B cells supplemented with exogenous recombinant ADA-2. (n=6 per group, in 2 independent experiments). (Wilcoxon, paired, nonparametric t-test (*p < 0.05). **(C-D)** Production of IL-6 and IL-2 in the supernatants of 5-days co-culture of GC-Tfh, pre-Tfh and non-Tfh subsets with their autologous GC B cells supplemented with exogenous recombinant ADA-2. (n=6 per group, in 2 independent experiments). (Wilcoxon, paired, nonparametric t-test (*p < 0.05); Mean±SEM)

Supplementary Figure 11

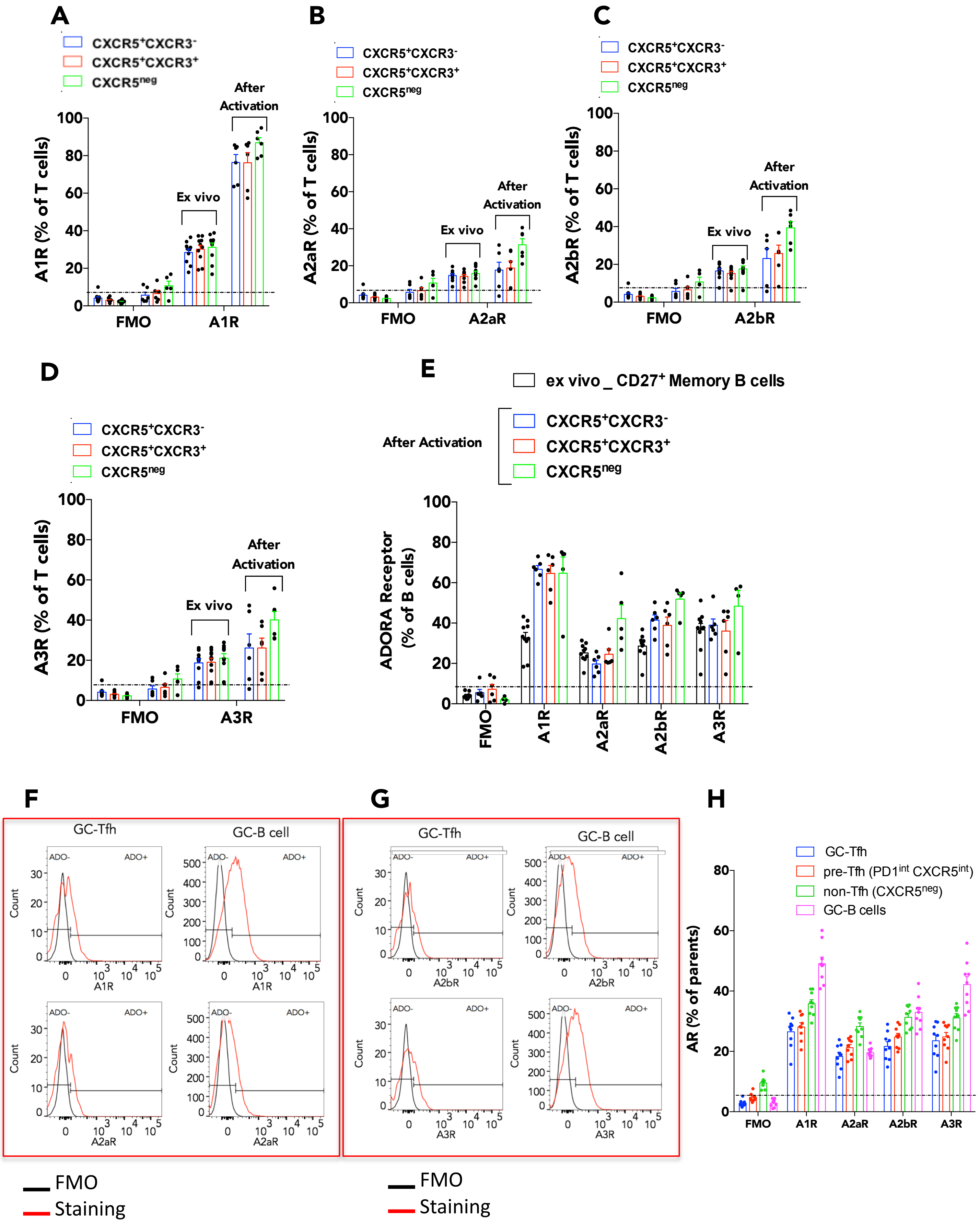

Mean±SEM

Supplementary Figure 11: *Ex vivo expression of ARs by cTfh, GC-Tfh and B cells subsets.*

Frequency of cTfh and non-cTfh subsets *ex vivo* (n=10 per group) and at 7-day co-culture (n=6 per group) expressing **(A)** A1R**,** **(B)** A2aR, **(C)** A2bR**,** and **(D)** A3R**.** **(E)** Frequency of memory B cells expressing adenosine receptors A1R, A2aR, A2bR and A3R in *ex vivo* (n=10 per group) and 7-day co-culture assays (n=6 per group). (FMO: Fluorescence minus one, i.e. anti-ARs antibodies)

(F) *Ex vivo* FACS profile expression of A1R (top), A2aR (bottom), and **(G)** A2bR (top) and A3R (bottom), respectively by GC-Tfh and GC-B cells **(H)** Frequency of *ex vivo* GC-Tfh, pre-Tfh, non-Tfh and GC-B subsets expressing A1R**,** A2aR, A2bR**,** and A3R**.** (n=9 per group). (FMO: Fluorescence minus one, i.e. anti-ARs antibodies) (Mean±SEM).

Supplementary Figure 12

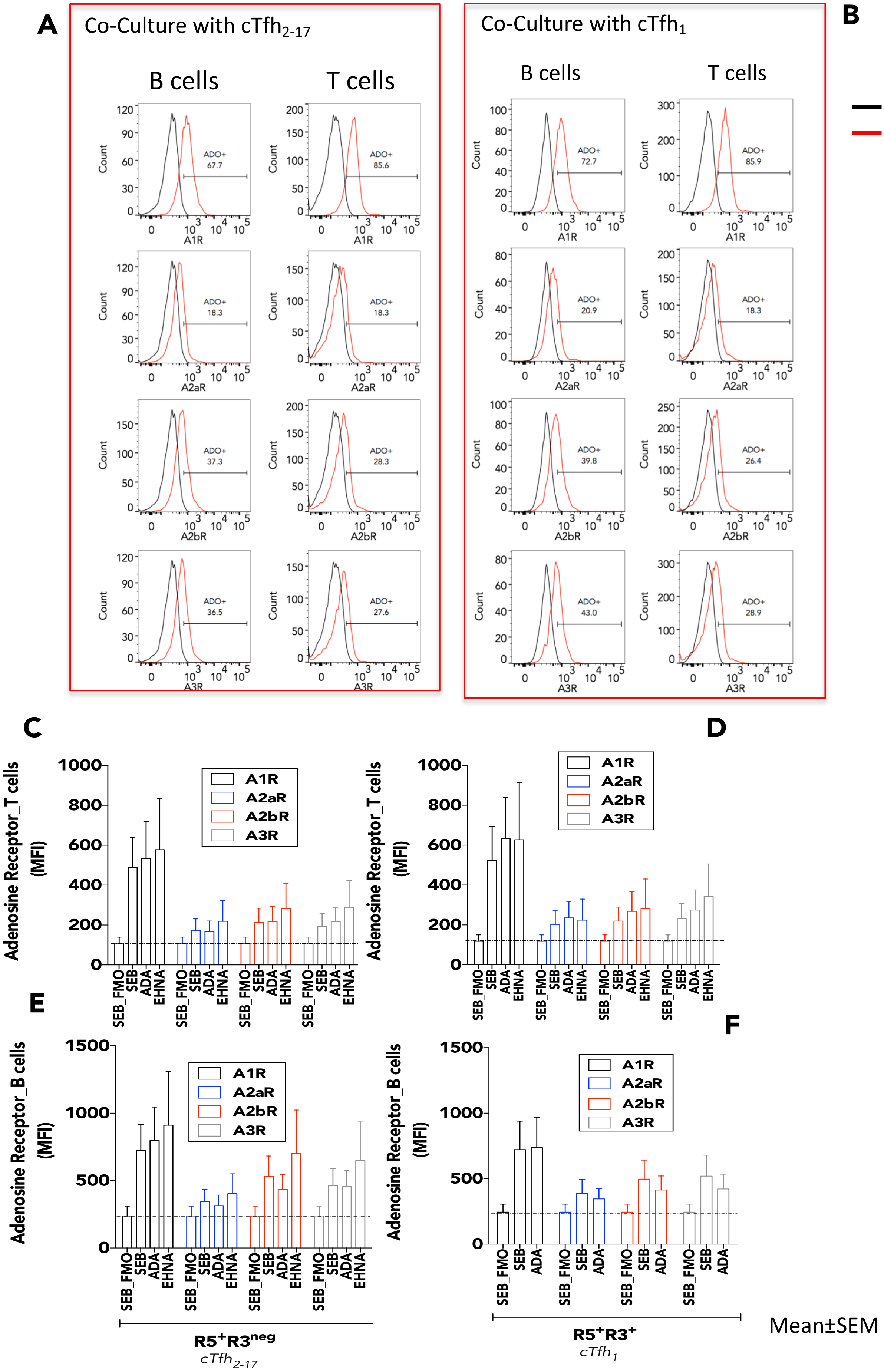

Supplementary Figure 12: *After co-culture expression of ARs by cTfh and autologous B cells subsets.*

FACS profile expression of A1R, A2aR, A2bR and A3R (top to bottom) by $cTfh_{2-17}$ **(A)**, $cTfh_1$ **(B)**, and respective co-cultured memory B cells after 7 days of incubation

(C) Frequency of $CXCR5^{+}CXCR3^{neg}$ $cTfh_{2-17}$ **(D)** $CXCR5^{+}CXCR3^{+}$ $cTfh_1$ expressing A1R**,** A2aR**,** A2bR and A3R after 7-day co-culture with memory B cells following treatment with ADA or its inhibitor. (n=5 per group, 2 independent experiment) **(E)** Frequency of co-cultured memory B cells with $CXCR5^{+}CXCR3^{neg}$ $cTfh_{2-17}$, or **(F)** with $CXCR5^{+}CXCR3^{+}$ $cTfh_1$ expressing A1R**,** A2aR**,** A2bR and A3R following treatment with ADA or its inhibitor EHNA (FMO: Fluorescence minus one, i.e. anti-ARs antibodies) (n=5 per group, 2 independent experiment) (Mean±SEM).

Supplementary Figure. 13

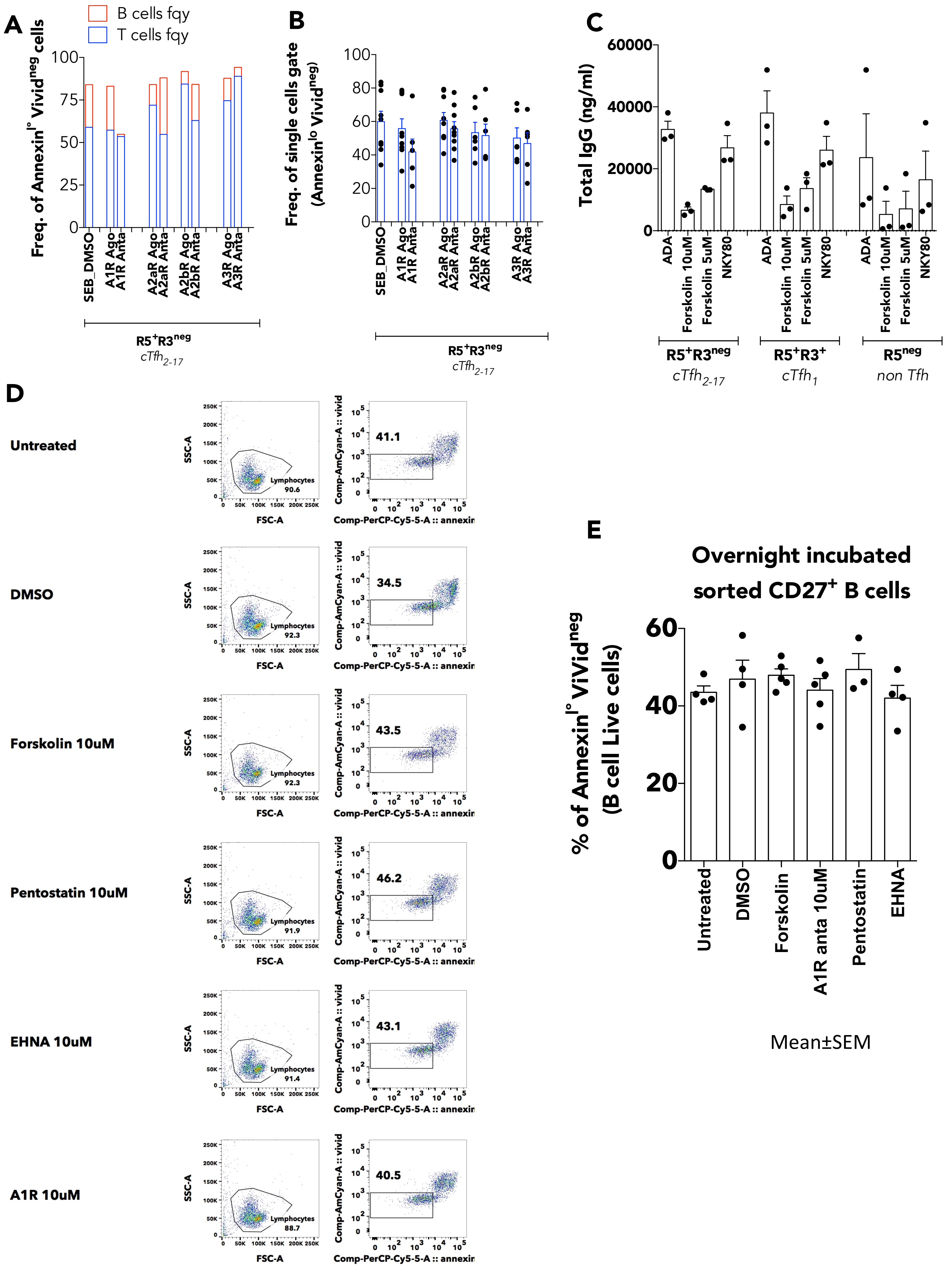

Supplementary Figure 13: *Compound biological effect is not due to toxicity.*

(A) Ratio of T vs B cells and **(B)** Frequency of live cells at day 7 co-culture of CXCR5^{+}CXCR3^{neg} cTfh$_{2\text{-}17}$ supplemented with or without individual adenosine receptor agonists or antagonists. **(C)** Production of IgG in the supernatants of co-culture assay with cTfh and non-cTfh subsets performed in the absence of SEB, but in presence of ADA-1 only, or supplemented with an activator (forksolin) (5 or 10 uM) or an inhibitor (NKY80) of adenylyl cyclase (AC). **(D)** Representative FACS plot from overnight culture assay of sorted memory B cells **(T cell free culture)** performed untreated or in the presence of DMSO, or of the activator of adenylyl cyclase (forksolin) (10 uM), or of the ADA inhibitors Pentostatin (10uM) or EHNA (10uM) or of the antagonist of A1R, showing non-apoptotic cells (Vivid^{lo}Annexin^{lo}). **(E)** Collection of 3-5 overnight culture assays of sorted memory B cells (T cell free culture) performed untreated or in the presence of DMSO, or of the activator of adenylyl cyclase (forksolin) (10 uM), or of the ADA inhibitors Pentostatin (10uM) or EHNA (10uM) or of the antagonist of A1R, compiling percentage non-apoptotic cells (Vivid^{lo}Annexin^{lo}). 10K cells have been used per well to mimic cell density of co-culture assays. (Mean±SEM)

Supplementary Figure 14

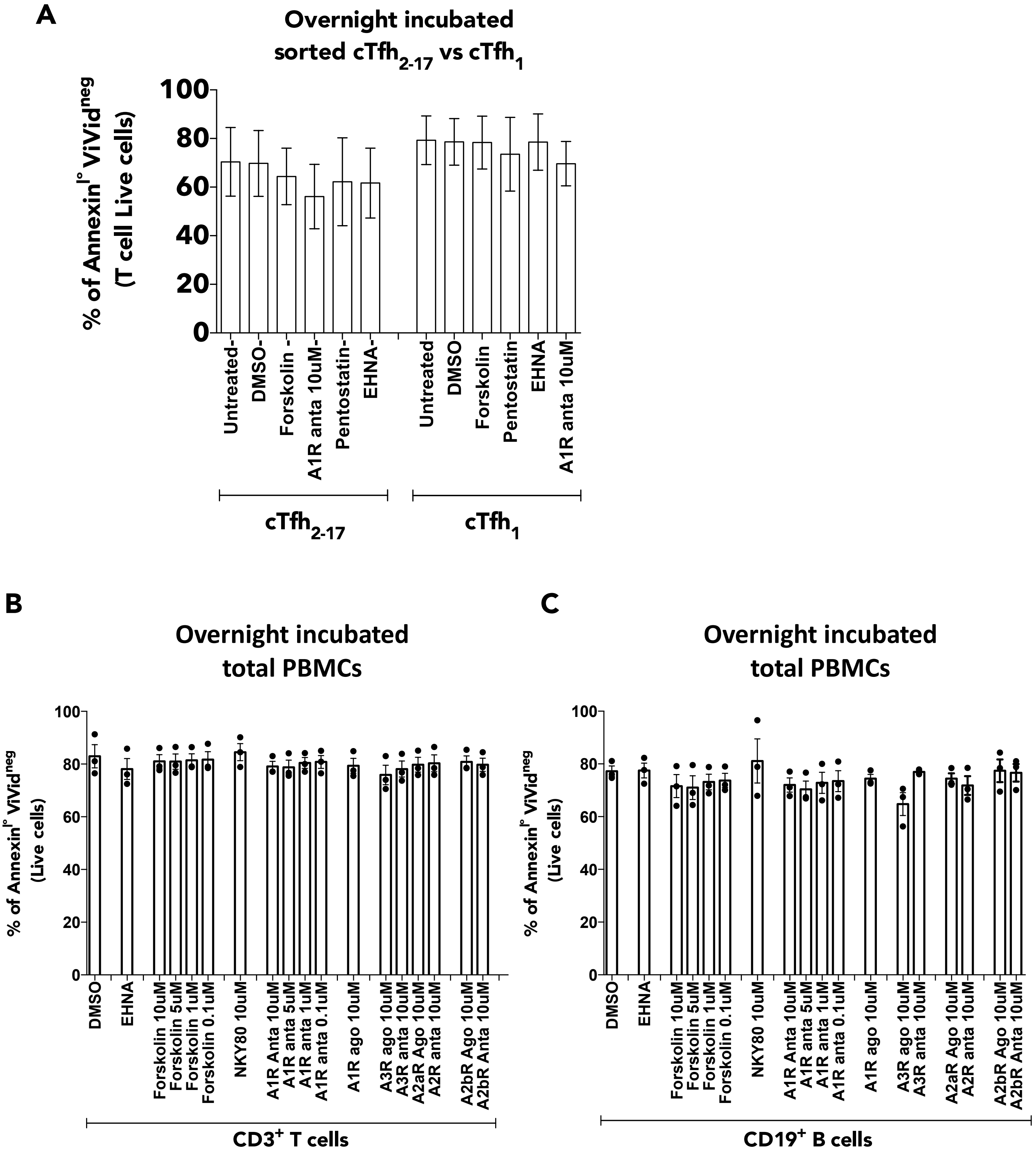

Mean±SEM

Supplementary Figure 14: *Compound biological effect is not due to toxicity.*

(A) Collection of 3-5 overnight culture assays of sorted $cTfh_{2-17}$ or $cTfh_1$ cells (B cell free culture) performed untreated or in the presence of DMSO, or of the activator of adenylyl cyclase (forksolin) (10 uM), or of the ADA inhibitors Pentostatin (10uM) or EHNA (10uM) or of the antagonist of A1R, compiling percentage of non-apoptotic cells ($Vivid^{lo}Annexin^{lo}$). 10K cells have been used per well to mimic cell density of co-culture assays. **(B)** Overnight culture assays of PBMCs performed in presence of the inhibitor of ADA (EHNA) or the activator of adenylyl cyclase (forksolin) (10-5-1-0.1 uM) or its inhibitor (NKY80) (10uM) or A1R antagonist (10-5-1-0.1 uM) or A1R agonist (10uM) or A3R agonist (10uM) or A3R antagonist (10uM) or A2aR agonist (10uM) or A2aR antagonist (10uM) or A2bR agonist (10uM) or A2bR antagonist (10uM); showing non apoptotic $CD3^+$T cells ($Vivid^{lo}Annexin^{lo}$). **(C)** Overnight culture assays of PBMCs performed in presence of the inhibitor of ADA (EHNA) or the activator of adenylyl cyclase (forksolin) (10-5-1-0.1 uM) or its inhibitor (NKY80) (10uM) or A1R antagonist (10-5-1-0.1 uM) or A1R agonist (10uM) or A3R agonist (10uM) or A3R antagonist (10uM) or A2aR agonist (10uM) or A2aR antagonist (10uM) or A2bR agonist (10uM) or A2bR antagonist (10uM); showing non apoptotic $CD19^+$B cells ($Vivid^{lo}Annexin^{lo}$) (Mean±SEM).

Supplementary Figure 15

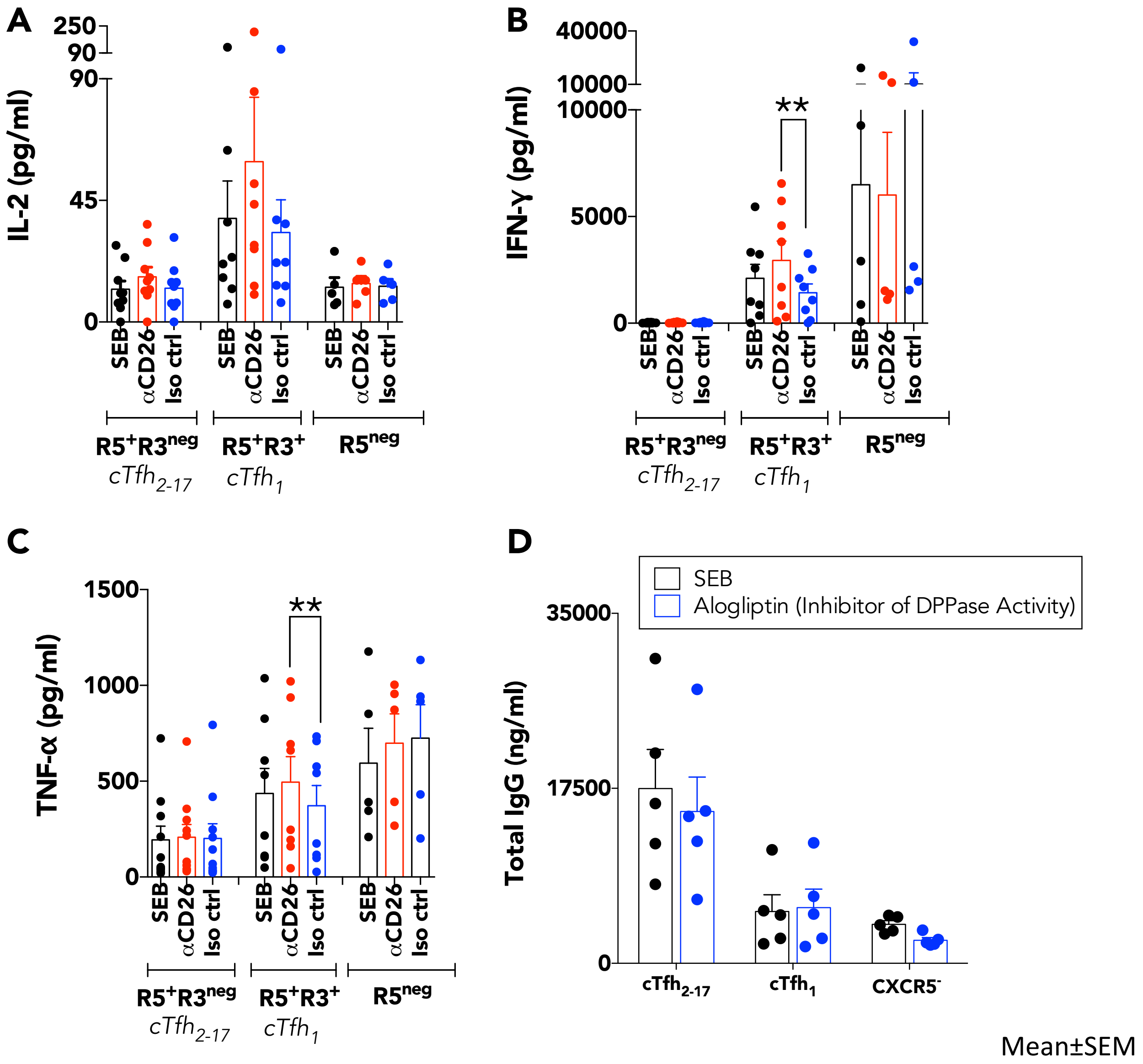

Supplementary Figure 15: *Cytokine baseline of the co-culture model from PBMCs supplemented with anti-human CD26.*

(A) Ratio of live (Annexin^{neg}) T vs B cells frequency at day 7 of co-culture of cTfh and non cTfh subsets supplemented with or without anti-human CD26 (134-2C2) or its isotype control (n=6, 2 independent experiment). **(B)** IL-6 **(C)** TNF-α **(D)** IL-2 secretion in the supernatants of co-culture of cTfh and non-cTfh subsets supplemented with or without anti-human CD26 (134-2C2) or its isotype control at day 7 (n=8 per group, 2 independent experiment). **(E)** Production of IgG in the supernatants of co-culture assay of cTfh and non-cTfh subsets supplemented with or without Alogliptin, known as an inhibitor of dipeptidyl-peptidase activity of CD26. (ANOVA, paired, nonparametric t-test (**p < 0.001), Mean±SEM).

Supplementary Figure 16

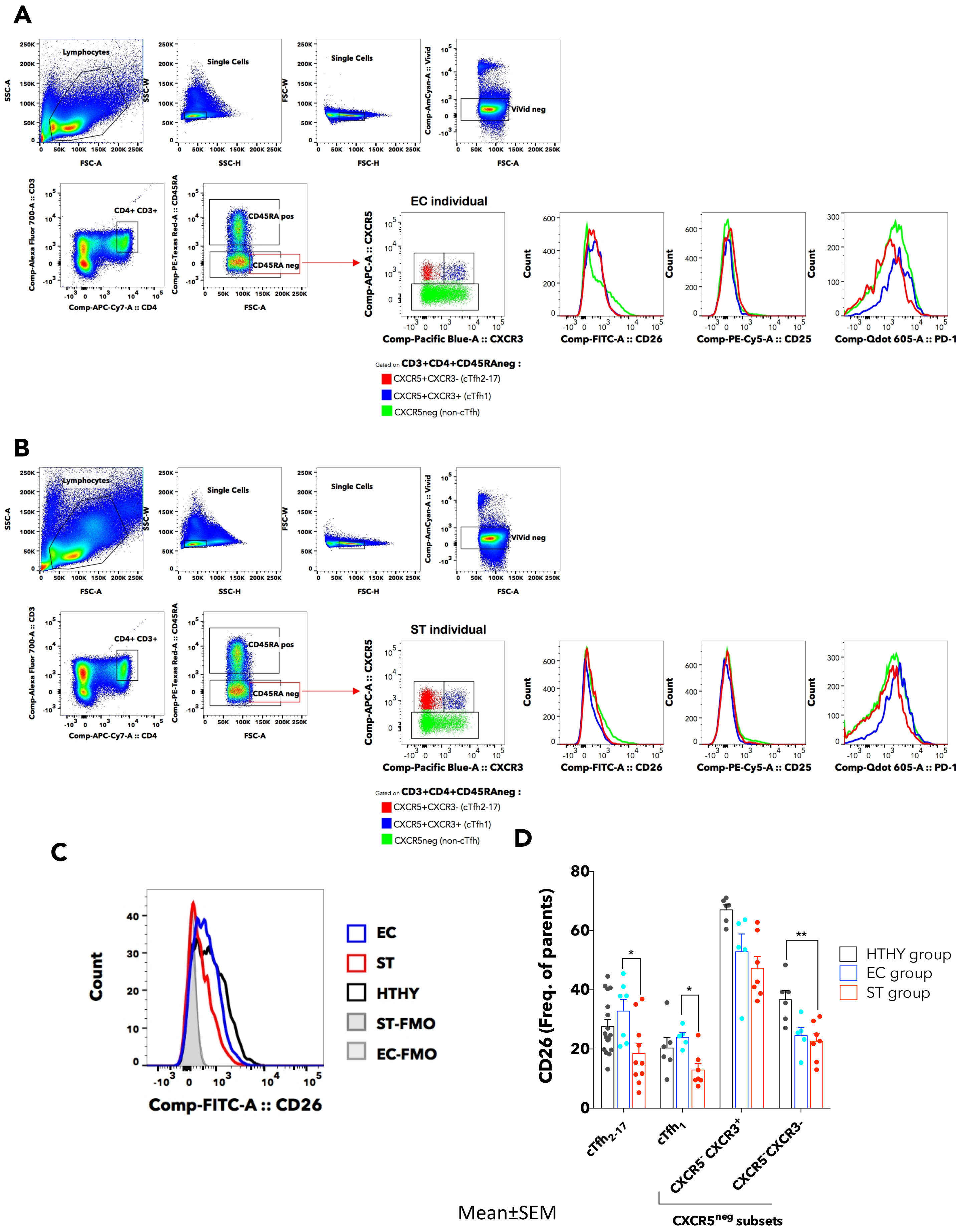

Supplementary Figure 16: *Ex vivo FACS representative profile of cTfh and CXCR5^{neg} subsets from HIV-infected patients*

(A) FACS representative profile of cTfh and CXCR5^{neg} subsets from EC patients. CD26, CD25 and PD1 expression are shown. cTfh$_{2-17}$ (red); cTfh$_1$ (blue) and CXCR5^{neg} T cells (green) subsets are overlaid for each marker. **(B)** FACS representative profile of cTfh and CXCR5^{neg} subsets from ST patients. CD26, CD25 and PD1 expression are shown. cTfh$_{2-17}$; cTfh$_1$ and CXCR5^{neg} T cells subsets are overlaid for each marker. **(C)** Representative FACS profile of CD26 overlays expression comparing EC, ST and HTHTY (healthy) individuals. **(D)** Compilation of *ex vivo* frequency of CD26$^+$ cells in each cTfh and non-cTfh subsets comparing comparing EC, ST and HTHTY (healthy) individuals. ST cTfh$_{2-17}$ and cTfh$_1$ express significantly less CD26 compared to EC or HTHY groups (ANOVA, nonparametric t-test (*$p < 0.05$; **$p < 0.001$) (Mean±SEM) .

Supplementary Table 1

Samples ID	Tissue Type	QC (Pass/Fail)	ADA-1 (H-score)	PDCD-1 (PD-1) (H-score)	Bcl-6 (H-score)
TSL077-a	Human Tonsil	PASS	175 (4x0+3x0+2x75+1x25)	75 (4x15+3x5+2x0+1x0)	285 (4x0+3x85+2x15+1x0)
TSL077-b			180 (4x0+3x0+2x80+1x20)	35 (4x5+3x5+2x0+1x0)	270 (4x0+3x70+2x30+1x0)
TSL077-c			150 (4x0+3x0+2x50+1x50)	55 (4x10+3x5+2x0+1x0)	250 (4x0+3x50+2x50+1x0)
TSL078-a		PASS	180 (4x0+3x0+2x80+1x20)	90 (4x15+3x10+2x0+1x0)	285 (4x0+3x85+2x15+1x0)
TSL078-b			190 (4x0+3x0+2x90+1x10)	110 (4x20+3x10+2x0+1x0)	290 (4x0+3x90+2x10+1x0)
TSL078-c			175 (4x0+3x0+2x75+1x25)	130 (4x25+3x10+2x0+1x0)	285 (4x0+3x85+2x15+1x0)
Note: All H-scores are based on follicle regions. Different follicles were selected on slides a-; -b and -c.					

Supplementary Table 2

Samples ID	Tissue Type	QC (Pass/Fail)	ADA-1 (H-score)	PDCD-1 (PD-1) (H-score)	Bcl-6 (H-score)
TSL077-a	Human Tonsil	PASS	35 (4x0+3x0+2x5+1x25)	29 (4x0+3x3+2x10+1x0)	40 (4x0+3x0+2x15+1x10)
TSL077-b			35 (4x0+3x0+2x5+1x25)	29 (4x0+3x3+2x10+1x0)	40 (4x0+3x0+2x15+1x10)
TSL077-c			35 (4x0+3x0+2x5+1x25)	29 (4x0+3x3+2x10+1x0)	40 (4x0+3x0+2x15+1x10)
TSL078-a		PASS	26 (4x0+3x0+2x3+1x20)	26 (4x0+3x0+2x3+1x20)	46 (4x0+3x1+2x10+1x25)
TSL078-b			26 (4x0+3x0+2x3+1x20)	26 (4x0+3x0+2x3+1x20)	46 (4x0+3x1+2x10+1x25)
TSL078-c			26 (4x0+3x0+2x3+1x20)	26 (4x0+3x0+2x3+1x20)	46 (4x0+3x1+2x10+1x25)
Note: All H-scores are based on non-follicle regions.					

Supplementary Table 1 and 2: *H-scoring details Tables*

(1) H-scoring inside the follicular area (3 slides per 2 samples).

(2) H-scoring outside the follicular area (3 slides per 2 samples).

Supplementary Table 3

Samples ID	Age	Diagnosis Year	ART duration (years)	Viral Load- Plasma HIV RNA-(copies/ml)	CD4 Count (cells/mm3) for LTNPor (cells/uL) for CA	CD8 Count (cells/mm3) for LTNPor (cells/uL) for CA	Status
L1	51	1989	NA	<50	913	519	LTNP
L4	50	2004	NA	<50	679	556	LTNP
L5	60	1985	NA	<50	1334	805	LTNP
L7	54	1985	NA	<50	902	722	LTNP
L8	55	1985	NA	<50	618	588	LTNP
L10	57	2007	NA	<50	1943	716	LTNP
LTNP17	52	2007	NA	<50	1943	716	LTNP
LTNP005	36	1999	NA	<50	711	574	LTNP
LTNP007	54	1995	NA	<50	711	574	LTNP
LTNP008	NA		NA	2772	514	555	LTNP
LTNP015	47	1991	NA	<50	611	583	LTNP
HAD-EC2	52	1995	NA	<50	552	607	LTNP
HAD-EC4	47	1997	NA	<50	712	260	LTNP
HAD-EC5	NA	2010	NA	<50	1225	977	LTNP
HAD-ST02	42	2003	2004	<40	779	1830	CA
HAD-ST03	53	1985	1988	<40	NA	NA	CA
HAD-ST11	52	2000	2000	<40	784	1173	CA
HAD-ST12	56	2006	2007	<40	NA	NA	CA
HAD-ST15	24	1992	2008	<40	623	657	CA
HAD-ST17	53	1997	1997	<40	848	1089	CA
HAD-ST18	48	1996	1998	<40	408	528	CA
HAD-ST21	33	2009	2009	<40	506	517	CA
HAD-ST34	NA	NA	NA	NA	NA	NA	CA
HAD-ST37	NA	NA	NA	NA	NA	NA	CA
HAD-ST39	34	2009	NA	<40	640	1474	CA
HAD-ST40	35	2002	NA	<40	679	922	CA
ST116	NA	NA	NA	NA	NA	NA	CA
ST118	50	NA	NA	<50	773	1130	CA

Supplementary Table 3 *Listing of EC (LTNP) and ST (CA) patients used for HIV study*
CA: Chronic Aviremic (ST); LNTP: Long-Term non progressor (EC); NA: Non-Applicable.
