# Supplementary material for: Adenosine deaminase-1 delineates human follicular helper T cell function and is altered with HIV
Source: Nat Commun. 2019 Feb 18;10:823. doi: 10.1038/s41467-019-08801-1 (PMC6379489; doi:10.1038/s41467-019-08801-1)
Supplement: Supplementary file 2 — Reporting Summary [file 41467_2019_8801_MOESM2_ESM.pdf]

nature research

Corresponding author(s): Elias K HADDAD

Last updated by author(s): Jan 15, 2019

# Reporting Summary

Nature Research wishes to improve the reproducibility of the work that we publish. This form provides structure for consistency and transparency in reporting. For further information on Nature Research policies, see Authors & Referees and the Editorial Policy Checklist.

## Statistics

For all statistical analyses, confirm that the following items are present in the figure legend, table legend, main text, or Methods section.

| n/a | Confirmed | |
|---|---|---|
| ☐ | ☒ | The exact sample size (*n*) for each experimental group/condition, given as a discrete number and unit of measurement |
| ☐ | ☒ | A statement on whether measurements were taken from distinct samples or whether the same sample was measured repeatedly |
| ☐ | ☒ | The statistical test(s) used AND whether they are one- or two-sided<br>*Only common tests should be described solely by name; describe more complex techniques in the Methods section.* |
| ☐ | ☒ | A description of all covariates tested |
| ☐ | ☒ | A description of any assumptions or corrections, such as tests of normality and adjustment for multiple comparisons |
| ☐ | ☒ | A full description of the statistical parameters including central tendency (e.g. means) or other basic estimates (e.g. regression coefficient) AND variation (e.g. standard deviation) or associated estimates of uncertainty (e.g. confidence intervals) |
| ☐ | ☒ | For null hypothesis testing, the test statistic (e.g. *F*, *t*, *r*) with confidence intervals, effect sizes, degrees of freedom and *P* value noted<br>*Give P values as exact values whenever suitable.* |
| ☒ | ☐ | For Bayesian analysis, information on the choice of priors and Markov chain Monte Carlo settings |
| ☒ | ☐ | For hierarchical and complex designs, identification of the appropriate level for tests and full reporting of outcomes |
| ☒ | ☐ | Estimates of effect sizes (e.g. Cohen's *d*, Pearson's *r*), indicating how they were calculated |

*Our web collection on statistics for biologists contains articles on many of the points above.*

## Software and code

Policy information about availability of computer code

| | |
|---|---|
| Data collection | N/A |
| Data analysis | N/A |

For manuscripts utilizing custom algorithms or software that are central to the research but not yet described in published literature, software must be made available to editors/reviewers. We strongly encourage code deposition in a community repository (e.g. GitHub). See the Nature Research guidelines for submitting code & software for further information.

## Data

Policy information about availability of data

All manuscripts must include a data availability statement. This statement should provide the following information, where applicable:

- Accession codes, unique identifiers, or web links for publicly available datasets
- A list of figures that have associated raw data
- A description of any restrictions on data availability

> Data that support the findings of this study have been deposited in GEO (Gene Expression Omnibus) with the accession codes GSE99782; hyperlink: https://www.ncbi.nlm.nih.gov/geo/query/acc.cgi?acc=GSE99782

> The authors declare that all other data supporting the findings of this study are available within the paper [and its supplementary information files].

# Field-specific reporting

Please select the one below that is the best fit for your research. If you are not sure, read the appropriate sections before making your selection.

☒ Life sciences ☐ Behavioural & social sciences ☐ Ecological, evolutionary & environmental sciences

For a reference copy of the document with all sections, see nature.com/documents/nr-reporting-summary-flat.pdf

# Life sciences study design

All studies must disclose on these points even when the disclosure is negative.

| | |
|---|---|
| Sample size | Samples are individual human sample. A minimum of 6 individuals (biological replicate) have been processed to perform each figure in a minimum of 2 independant experiments (technical replicate) in order to reach statistical significance. |
| Data exclusions | No data were excluded. |
| Replication | A minimum of 6 individuals (biological replicate) have been processed to perform each figure in a minimum of 2 independant experiments (technical replicate) in order to reach statistical significance. |
| Randomization | Sample were allocated randomly to experimental groups, except when purpose of the experiment compare elite controler vs chronic aviremic patients. |
| Blinding | H-score comparing follicle vs. non follicle ADA-1expression were blindly obtained, because performed by an independant company (Advanced Cell Diagnostics) |

# Reporting for specific materials, systems and methods

We require information from authors about some types of materials, experimental systems and methods used in many studies. Here, indicate whether each material, system or method listed is relevant to your study. If you are not sure if a list item applies to your research, read the appropriate section before selecting a response.

## Materials & experimental systems

| n/a | Involved in the study | |
|---|---|---|
| ☐ | ☒ | Antibodies |
| ☒ | ☐ | Eukaryotic cell lines |
| ☒ | ☐ | Palaeontology |
| ☒ | ☐ | Animals and other organisms |
| ☐ | ☒ | Human research participants |
| ☒ | ☐ | Clinical data |

## Methods

| n/a | Involved in the study | |
|---|---|---|
| ☒ | ☐ | ChIP-seq |
| ☐ | ☒ | Flow cytometry |
| ☒ | ☐ | MRI-based neuroimaging |

## Antibodies

| | |
|---|---|
| Antibodies used | Biolegend: CD3 (HIT3α) (dilution: 1/100; Cat. Number: 300324), CD4 (RPA-T4) (dilution: 1/200; Cat. Number: 300518), CD25 (BC96) (dilution: 1/100; Cat. Number: 302608), CD27 (O323) (dilution: 1/100; Cat. Number: 302829), CD38 (HIT2) (dilution: 1/50; Cat. Number: 302532), PD-1 (EH12.2H7) (dilution: 1/100; Cat. Number: 329918 or 329930), CXCR3 (G025H7) (dilution: 1/200; Cat. Number: 353716), CD319 (162.1) (dilution: 1/100; Cat. Number: 331806), CD26 (BA5b) (dilution: 1/100; Cat. Number: 302704), IgD (clone IA6-2) (dilution: 1/100; Cat. Number: 348226), IL7-R (A019D5)(dilution: 1/100; Cat. Number: 351309) and CD19 (HIB19) (dilution: 1/100; Cat. Number: 302216 or 302226)<br>BD Biosciences: CXCR5 (RF8B2) (dilution: 1/20; Cat. Number: 562781), Bcl-6 (clone K112-91) (dilution: 1/20; Cat. Number: 561522) , Annexin V (dilution: 1/200; Cat. Number: 561431)<br>Beckman Coulter: CD45RA (2H4LDH11LDB9) (dilution: 1/200; Cat. Number: IM2711U)<br>Biorbyt, UK: Anti-human adenosine receptor antibodies (dilution: 1/50), i.e., ADORA1 antibody (Cat. Number: orb102041), ADORA2A (Cat. Number: orb15053), ADORA2b (Cat. Number: orb102042), ADORA3 (Cat. Number: orb15062) |
| Validation | Each antibody has been validated by the companies and by the result of the paper, where controls including FMO are provided. |

## Human research participants

Policy information about studies involving human research participants

| | |
|---|---|
| Population characteristics | Population are young healthy individual, HIV elite controler - long term non progressor and chronic aviremic patients. |

| | |
|---|---|
| Population characteristics | HIV patients details are described into the supplementary table 3. |
| Recruitment | Blood samples and tonsils from healthy donors, HIV-infected elite controllers (ECs) and HIV-infected chronically treated aviremic subjects (CA) were obtained from Martin Memorial Health Systems (Florida), the Clinical Research Center, National Institutes of Health (NIH) and the Chronic Viral Illness Service at McGill University Health Centre. |
| Ethics oversight | The Institutional Review Boards at the relevant institutions approved all procedures, and all participants provided signed informed consent. |

Note that full information on the approval of the study protocol must also be provided in the manuscript.

# Flow Cytometry

## Plots

Confirm that:

☒ The axis labels state the marker and fluorochrome used (e.g. CD4-FITC).

☒ The axis scales are clearly visible. Include numbers along axes only for bottom left plot of group (a 'group' is an analysis of identical markers).

☒ All plots are contour plots with outliers or pseudocolor plots.

☒ A numerical value for number of cells or percentage (with statistics) is provided.

## Methodology

| | |
|---|---|
| Sample preparation | Samples are thawed in complete RPMI and stained into sorting buffer, which is non red-phenol RPMI + 2% FBS + 10% PS + 10% Glutamine and 2,5% Hepes or FACS buffer, which is PBS + 2% FBS. Because of the cell population abundance, multiple vials of PBMCs (from same patient) are pooled and enriched for T or B cells before sorting to shorten sorting time. |
| Instrument | For sorting: BD™ FACS Aria Fusion or BD™ FACSAria III<br>For flow cytometry acquisition: BD™ FACS Fortessa |
| Software | Data were acquired with software BD FACSDIVA and analyzed with FlowJo v10. |
| Cell population abundance | All those cell subset (cTfh, GC-Tfh, GC-B cells) represent less than 10% of total cells. |
| Gating strategy | > CXCR5+CXCR3+CCR6+/- cTfh2-17 ; CXCR5+CXCR3+CCR6+/- and non Tfh CXCR5neg cells subsets, used in the PBMCs co-culture model, are gated on lived CD3+CD4+CD45RAneg and separated by CXCR5 and CXCR3 staining strategy.<br>> Memory B cells, used in the co-culture assay, are gated out on lived CD19+CD27+ cells<br>> CXCR5hiPD-1hi (GC-Tfh), CXCR5intPD-1int (pre-Tfh/Tfh), CXCR5neg subsets, used in the tonsillar co-culture model, are gated lived CD3+CD4+CD45RAneg and separated by CXCR5 and PD-1 staining strategy. Of note, each T cell population has been sorted from CD25neg population<br>> GC-B cells, used in the tonsillar co-culture assay, are gated out on lived CD19+ B cells and separated by IgD and CD38 staining strategy. GC-B cells are IgDnegCD38intCD319loBcl6+ |

☒ Tick this box to confirm that a figure exemplifying the gating strategy is provided in the Supplementary Information.
